# Similar burden of pathogenic coding variants in exceptionally long‐lived individuals and individuals without exceptional longevity

**DOI:** 10.1111/acel.13216

**Published:** 2020-08-29

**Authors:** Danielle Gutman, Gabriel Lidzbarsky, Sofiya Milman, Tina Gao, Patrick Sin-Chan, Claudia Gonzaga‐Jauregui, Regeneron Genetics Center, Joris Deelen, Alan R. Shuldiner, Nir Barzilai, Gil Atzmon

**Affiliations:** ^1^ Faculty of Natural Sciences University of Haifa Haifa Israel; ^2^ Department of Medicine Albert Einstein College of Medicine Bronx New York USA; ^3^ Regeneron Genetics Center Tarrytown New York USA; ^4^ Max Planck Institute for Biology of Ageing Cologne Germany; ^5^ Molecular Epidemiology Department of Biochemical Data Sciences Leiden University Medical Center Leiden The Netherlands; ^6^ Genetic, Institute for Aging Research and the Diabetes Research Center Albert Einstein College of Medicine Bronx New York USA

## Abstract

Centenarians (exceptionally long‐lived individuals—ELLI) are a unique segment of the population, exhibiting long human lifespan and healthspan, despite generally practicing similar lifestyle habits as their peers. We tested disease‐associated mutation burden in ELLI genomes by determining the burden of pathogenic variants reported in the ClinVar and HGMD databases using data from whole exome sequencing (WES) conducted in a cohort of ELLI, their offspring, and control individuals without antecedents of familial longevity (*n* = 1879), all descendent from the founder population of Ashkenazi Jews. The burden of pathogenic variants did not differ between the three groups. Additional analyses of variants subtypes and variant effect predictor (VEP) biotype frequencies did not reveal a decrease of pathogenic or loss‐of‐function (LoF) variants in ELLI and offspring compared to the control group. Case–control pathogenic variants enrichment analyses conducted in ELLI and controls also did not identify significant differences in any of the variants between the groups and polygenic risk scores failed to provide a predictive model. Interestingly, cancer and Alzheimer's disease‐associated variants were significantly depleted in ELLI compared to controls, suggesting slower accumulation of mutation. That said, polygenic risk score analysis failed to find any predictive variants among the functional variants tested. The high similarity in the burden of pathogenic variation between ELLI and individuals without familial longevity supports the notion that extension of lifespan and healthspan in ELLI is not a consequence of pathogenic variant depletion but rather a result of other genomic, epigenomic, or potentially nongenomic properties.

## Graphical abstract

The burden of disease‐causing mutation is similar among exceptionally long‐lived individuals, their offspring, and people with no familial exceptional longevity. Our results suggest the favorable longevity phenotype is an outcome of genetic buffers.

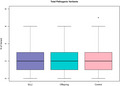

## 1 INTRODUCTION

Exceptionally long‐lived individuals (ELLI) are a unique segment of the population who exhibit not only long human lifespan but also long healthspan, and seemingly often overcome the adverse environmental effects on their physiological health (Ismail et al., 2016; Milman & Barzilai, 2015; Sebastiani et al., 2013). For this reason, they represent an extreme phenotype of successful aging (Tesi et al., 2018). The prevalence of centenarians is estimated to be approximately 1/3000 individuals in the United States (US) and Europe (Teixeira, Araújo, Jopp, & Ribeiro, 2017), and this rare group is studied all around the globe (Nebel & Schreiber, 2004; Teixeira et al., 2017) with the aim of identifying the biological mechanisms for healthy aging.

Exceptional longevity and healthy aging were shown to be hereditary in many familial studies (Atzmon et al., 2010; Beekman et al., 2013; Brooks‐Wilson, 2013; Erikson et al., 2016). First‐degree relatives of ELLI, including their offspring, demonstrate longer lifespan and decreased susceptibility to age‐related diseases, such as cardiovascular disease, dementia, and cancer, compared to the general population (Atzmon et al., 2010; Balistreri et al., 2014; Barzilai, Gabriely, Gabriely, Iankowitz, & Sorkin, 2001; Gubbi et al., 2017; Sebastiani, Nussbaum, Andersen, Black, & Perls, 2015). However, the genetic mechanisms facilitating the hereditary advantage have not yet been firmly established. Although a few longevity‐associated genetic signatures and individual gene variants have been identified, fewer have been replicated (Broer et al., 2014; Deelen et al., 2011; Joshi et al., 2016; Pilling et al., 2016; Sebastiani et al., 2012). Several studies have noted that ELLI may carry pathogenic mutations that increase the risk for cancer or Alzheimer's disease (Freudenberg‐Hua et al., 2014; Holstege et al., 2014; Stevenson et al., 2015; Tindale et al., 2015). These observations have led to the hypothesis that ELLI carry protective gene variants that “buffer” the effects of pathogenic variants (Bergman, Atzmon, Ye, MacCarthy, & Barzilai, 2007). Interestingly, somatic mutations are also known to accumulate with age (Milholland, Auton, Suh, & Vijg, 2015; Ye et al., 2013), challenging the physiological homeostasis and relative health observed in ELLI. Consequently, it could be hypothesized that one would expect to find a higher number of pathogenic variants in ELLI than in unrelated controls. A contradicting hypothesis is that ELLI possess "the perfect genome," containing a lower burden of pathogenic variation compared to the general population (Freudenberg‐Hua et al., 2016; Milman & Barzilai, 2015; Stevenson et al., 2015; Ye et al., 2013). Both hypotheses require gathering of additional evidence in support or contradiction of them.

We aimed to test the hypothesis of whether the ELLI genomes are relatively depleted of coding pathogenic variants compared to individuals without genetic predisposition to exceptional longevity in a cohort of ELLI, offspring of ELLI, and unrelated controls without familial longevity, using a cohort (differing from the above mentioned) from a founder population of Ashkenazi Jews (Table 1). Using a population with a strong founder effect increases statistical power to identify genetic factors responsible for traits of interest (Carmi et al., 2014; Freudenberg‐Hua et al., 2014; Lencz et al., 2018). The Ashkenazi Jewish population in the United States is among the largest founder populations in the world, and as such, it has substantial potential for natural variation and offers sufficient genetic and phenotypic diversity (Carmi et al., 2014; Lencz et al., 2018). The coding disease‐associated variants that were investigated were sourced from two well‐established databases, ClinVar (Landrum et al., 2017), and the Human Gene Mutation Database (HGMD^®^) (Stenson et al., 2017). ClinVar is a publicly available database that compiles and aggregates interpretations of clinically relevant genetic variants. It is one of the largest publicly available databases for clinically relevant variation and provides a reliable and updated source for analyses of pathogenic variation burden in genomic samples. HGMD is a curated commercial database that catalogues genetic variation reported as associated with human diseases. We chose to assess pathogenic variants since it was demonstrated that higher burden of disease‐associated variants is correlated with higher disease risk (Bick et al., 2012; Milholland et al., 2015; Patel et al., 2017). Together, the variants from both databases comprise a comprehensive list of pathogenic variants and were used to assess, using various approaches, the difference in disease‐causing mutation load (defined as the amount of potentially harmful mutations per individual) between the three groups in our cohort.

**Table 1:** Group age information

Group	Mean age	SD	Min age	Max age
Control	74	8.7	43	94
Offspring	70	7.87	43	94
ELLI	97.7	3.43	95	110

## 2 RESULTS

### 2.1 Annotation of pathogenic exome variants

**Figure 1:**
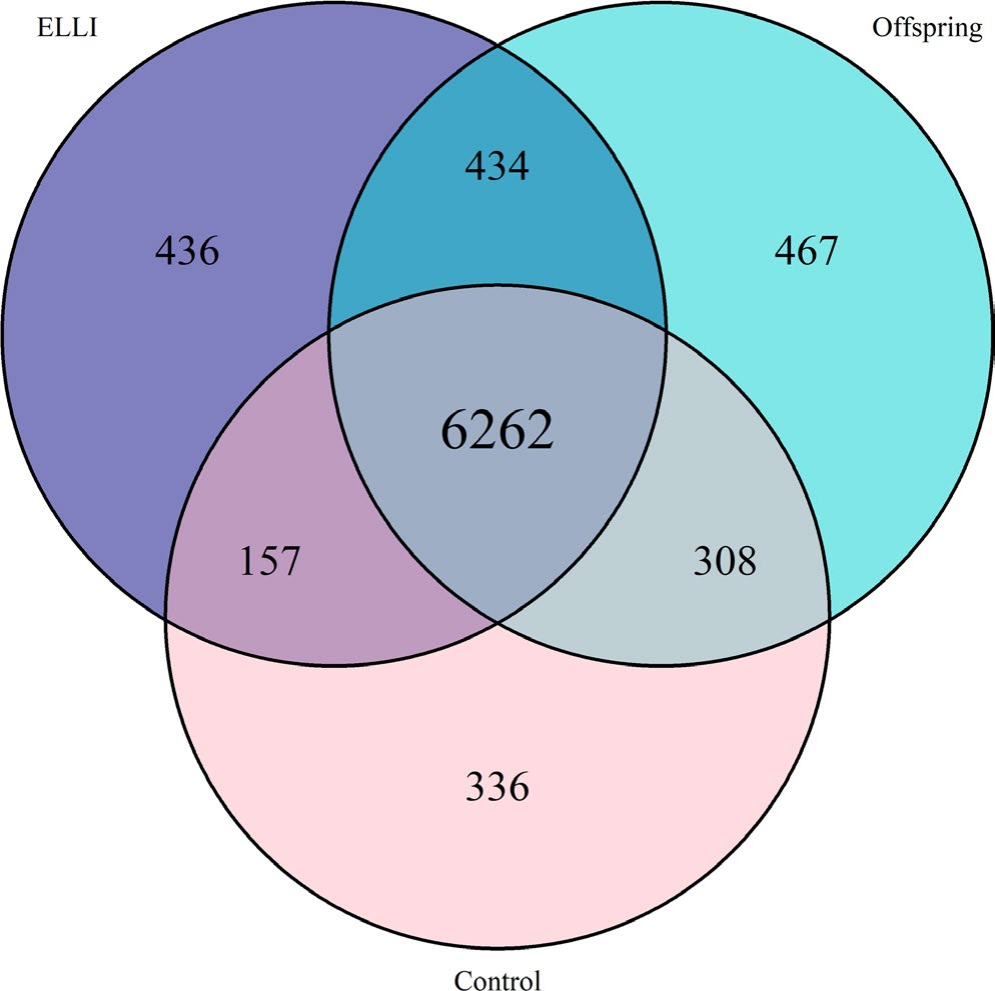
Pathogenic Variants. Venn diagram showing the number of pathogenic variants present in each group and in the unions between them

A total of 777,023 coding variants (623,003 in ELLI, 656,599 in offspring, and 609,864 in controls, Figure S1) passed the QC stage (Figure S2) and were queried using the compiled list of disease‐associated variants. The dispersion of the groups was homogenous as can be seen in Figure S3. The three groups had a large portion of variants in common. Among all the variant identified 64.9% (504,861) of the variants were shared between all 3 groups and among the pathogenic variants annotated 74.5% (6262) of the variants were shared between all 3 groups (Figure S1 and Figure 1, diagrams generated using VennDiagram R package (62)). The total number of variants recognized by Ensemble VEP was 7288 in ELLI, 7470 in offspring, and 7062 in controls. The distributions of variants by biotype and coding consequences were very similar between the cohort groups (Figures S4 and S5). The frequencies of VEP biotypes also did not differ by group (Figure S8).

### 2.2 Case–control association analysis

In order to identify candidate pathogenic variants that were differentially enriched in any of the groups, we performed case–control association tests. These tests did not reveal any variants that were significantly associated with ELLI status (Figures S3, [Link], [Link], [Link], [Link] and Table S1).

### 2.3 Mutation load

**Figure 2:**
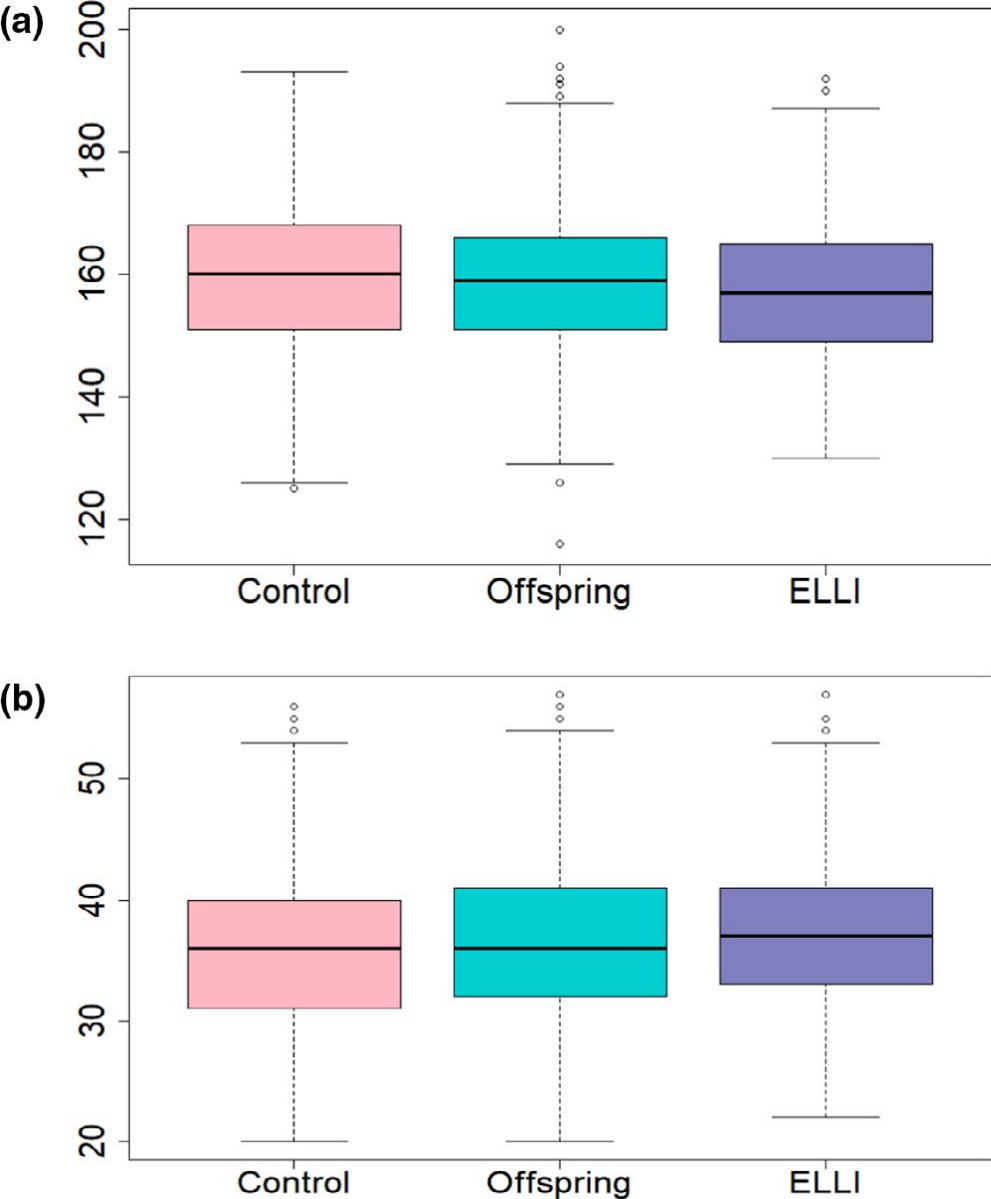
Comparison of age‐associated disease variants in the 3 groups. The bold horizontal line in each box represents the median value of individual age‐associated disease variants in the respective distribution. The area between the top and bottom lines is the IQR (a) Heterozygous age‐associated disease variants per individual by group. (b) Homozygous age‐associated disease variants per individual by group

The median mutation load per individual of all pathogenic variants (Table S2) was not significantly different between the three groups for both heterozygous and homozygous variants (KW *p* = 0.2 and 0.29, for hetero‐ and homozygous variants, respectively, Table S3, Figure 2 and Figure S9).

**Figure 3:**
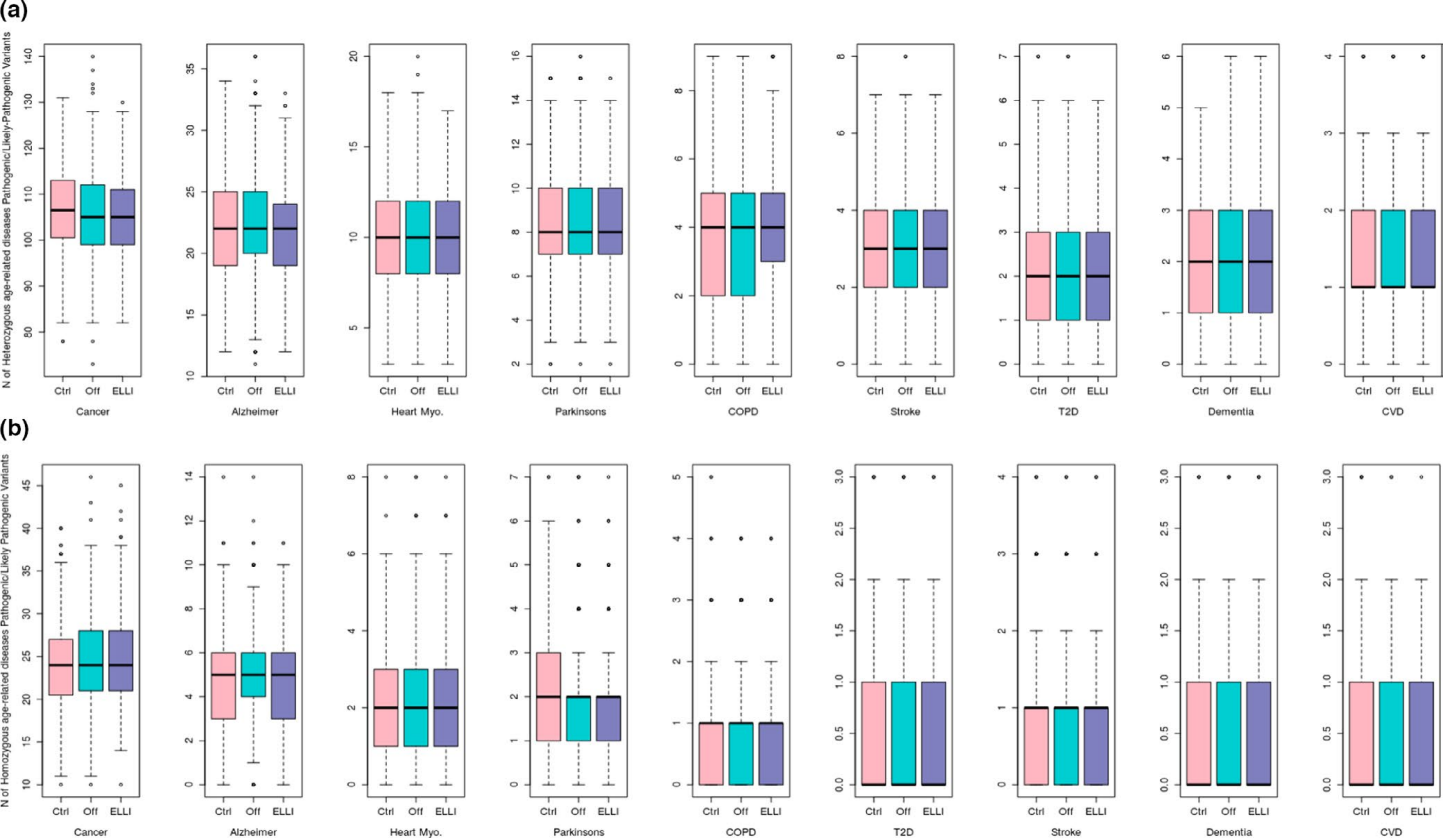
Disease‐related pathogenic variants in each group. The bold horizontal line in each box represents the median value of individual disease‐associated variants in the respective distribution. The area between the top and bottom lines is the IQR. (a) Heterozygous variants per individual by group by disease. (b) Homozygous variants per individual by group by disease

Interestingly, the difference between ELLI and controls was significant for common heterozygous age‐associated disease susceptibility variants (KW *p* = 0.009, Dunn test *p* = 0.0046) with controls carrying slightly more such variants (160 vs. 157 per individual, Table S3, Figure 2 and Figure S9). Analysis of disease‐specific susceptibility variants categorized by disease type revealed that the ELLI carried a significantly lower burden of heterozygous variants for cancer and Alzheimer's disease (KW *p* = 0.027 for cancer and 0.019 for Alzheimer's, and Dunn test *p* = 0.014 between ELLI and control and *p* = 0.008 between ELLI and offspring, Table S4 and Figure 3), although the mean numbers of variants are very similar (106 vs. 105 heterozygous variants for cancer and 22 vs. 21 in Alzheimer's for control and ELLI respectively). No significant differences in mutation load were noted for the remainder of age‐associated disease susceptibility variants between ELLI and controls. Further, using the strict filtering of pathogenic 2* ClinVar variants and HGMD high confidence disease‐causing variants (Table S5) did not yield any significant differences between the groups (Table S3 and Figure S10). An additional categorization of the strict filtering into autosomal recessive (AR), autosomal dominant (AD), and both autosomal recessive and dominant (AR/AD) modes of inheritance did not highlight any differences between the groups either (Table S6). These results did not vary by gender.

### 2.4 Variant effect predictions, eQTL characterization, and polygenic risk scores

MAFs (Minor Allele Frequency) were evaluated in order to assess the frequency of rare variants between the three groups, revealing no statistically significant differences between ELLI, offspring, and controls (Figures S11 and S12). To gain deeper biological insights alluding to possible molecular function of variants, variant effect predictions and biotypes were queried and found almost identical between the three groups (Figure S5). The pathogenic variants that were present in the eQTL collections also did not show significant differences between the three groups with 1192 shared eQTL positions and only 1‐4 unique variants per group in the SCAN database, and 949 shared and 0‐1 unique positions in the GRASP database. Polygenic risk scores showed low predictive value of SNPs in our data set. The highest R2 values were all below 0.025 indicating very low predictive value for the longevity phenotype (Figures [Link], [Link], [Link] and Table S7).

## 3 DISCUSSION

ELLI are a group of special interest due to their unique phenotype that is characterized by exceptional longevity and frequently preserved good health (Andersen, Sebastiani, Dworkis, Feldman, & Perls, 2012; Puca, Spinelli, Accardi, Villa, & Caruso, 2018; Sebastiani et al., 2015). In an effort to explore factors that may be responsible for these unique characteristics, we conducted a study to test whether the genomes of ELLI are depleted, or not, of pathogenic variants compared to individuals without familial exceptional longevity. For this purpose, we analyzed whole exome sequencing (WES) data in order to examine functional coding variants and focus on pathogenic variants from two established databases. In accordance with other studies (Freudenberg‐Hua et al., 2014; Holstege et al., 2014; Stevenson et al., 2015; Tindale et al., 2015), we identified many pathogenic variants among the ELLI. Further, our results also indicate that the ELLI carry a similar burden of pathogenic variation compared to control individuals from the same population without exceptional longevity. The similarity in amounts of variants was striking to us, especially given the expectation of somatic mutation accumulation previously reported (Milholland et al., 2015; Ye et al., 2013). Keeping in mind the chronological age gap between our cohort groups, a similar mutation accumulation between ELLI and controls suggests a different aging rate for the ELLI. Very low polygenic risk scores, obtained using longevity as the trait tested, indicate no predictive value and elude away from a gene coding interaction underlying the exceptional longevity phenotype. With 490–503 variants included in the analyses, there was no prediction of the longevity phenotype among our functional variants. These findings suggest that exceptional lifespan and healthspan are not attributable to a relative depletion of pathogenic gene variants.

Noteworthy are two significant differences we observed. (1) When looking into specific age‐associated diseases, we found that the ELLI group carried less pathogenic variants associated with cancer and with Alzheimer's disease, in contrast to our expectations based on Milholland et al. (2015) and Ye et al. (2013). This result is intriguing in light of the vast evidence linking somatic mutation accumulation and those two age‐associated diseases (Dapeng, Wang, & Di, 2016; Lodato & Walsh, 2019; Milholland et al., 2015; Park et al., 2019). Further, it is possible that the mutation accumulation in our ELLI group is slower than the accumulation in the control group; however, this rate was not examined in this study. That said, the similarity in amounts of pathogenic variants and the specific significance in difference in the cancer and Alzheimer's disease variants hint at this and can provide a lead for a follow‐up study.

In the context of other findings, this result is not surprising. A smaller study based on whole genome sequencing of 44 ELLI from our cohort identified 130 “Pathogenic/Likely Pathogenic” coding variants (Freudenberg‐Hua et al., 2014). A similar observation was reported by Stevenson et al. who investigated disease variants' burden in the Long Life Family Study and found no significant differences in the genetic risk for major age‐associated diseases among ELLI participants, the ELLI's offspring, and the offspring's spouses, who served as a control group (Stevenson et al., 2015). These results are also consistent with another study that characterized the whole exome of a pair of ELLI brothers and did not find any significant difference between them and the population genome (Tindale et al., 2015). The consistency of our results with these studies steers away from the "perfect genome" hypothesis.

The findings of this study support phenotypic and lifestyle studies performed by us and others, describing a slower aging rate in our unique cohort. Since ELLI maintain their healthspan and lifespan despite a similar burden of pathogenic germline variants compared to individuals without familial longevity, then it is possible that their genomes could be enriched for protective gene variants or regulatory variation that buffer the negative impact of pathogenic variants. This theory was proposed by Bergman et al. (2007), with support for it accumulating. While the “buffer” effect tones down the effect of pathogenic variation, it does not modulate the accumulation of mutation. Thus, we believe that in ELLI genomes the rate of mutation accumulation slows down, resulting in a similar amount of pathogenic mutations between ELLI and controls. We recently established a younger DNA methylation profile in ELLI in a smaller cohort of ELLI and unrelated controls, demonstrating DNA methylation clocks under‐estimating the phenotypic age of ELLI, supporting the slower rate of the aging process in ELLI (Gutman et al., 2020). This hypothesis requires additional in depth assessment and testing, not presented in our current study.

The use of whole exome sequencing data for this analysis allowed for the comparison of both common and rare functional coding variants in a large cohort of ELLI, offspring, and controls from the same founder population, strengthening the genetic homogeneity of this study. Thus, the lack in pathogenic variant differences between the study groups is unlikely to be confounded by differences among ELLI and controls. Additionally, focusing on the “Pathogenic/Likely Pathogenic” ClinVar variants together with HGMD variants and further subjecting these to annotation by the Enselmbl VEP, allowed us to screen for the genetic variants of greatest clinical relevance potential while considering mode of inheritance, yet no differences between ELLI and controls were identified. The unpredictive polygenic risk scores show absence of polygenic interactions in this phenotype; hence, we suggest possibly investigating noncoding interactions next. Despite the many strengths of our study, one limitation of our analysis is due to the fact that we focused on sequencing of the coding regions, and thus, other functional regions, such as flanking and intronic regions that may contain genetic regulatory elements, cannot be analyzed for variation differences. These should be explored in future studies that utilize whole genome sequencing for thorough investigations of regulatory genomic regions and the variance of those regions across similar cohorts. Additionally, the presence and prevalence of the disease‐associated phenotypes in relationship to the identified pathogenic variants in our cohort should be further characterized.

The lack of significant differences in the burden of pathogenic gene variants between the ELLI and controls does not support the notion that ELLI have a “perfect” genome that is depleted of pathogenic variants. Some significant values in heterozygous somatic cancer and Alzheimer's disease‐associated variants that suggest a slower rate of mutation accumulation are intriguing but not sufficient to explain the phenotypic differences. However, as our approaches and methods at predicting pathogenic variants advance, it may become necessary to revisit this question again in the future. With the ever‐increasing interest and knowledge in epigenetics and gene–gene interactions, these concepts should be further pursued as well, in order to gain better insights and understanding of the genetic underpinnings of the aging process.

## 4 EXPERIMENTAL PROCEDURES

### 4.1 Study population

DNA samples from 515 ELLI (mean age = 97.7 years old, range 95–110), 832 offspring of ELLI (offspring), and 532 controls (without familial longevity) all of Ashkenazi Jewish descent (described in Table 1), were collected as part of the Longevity Genes Project and the LonGenity studies at the Albert Einstein College of Medicine as previously described (Barzilai et al., 2001; Gubbi et al., 2017). The studies were approved by the Institutional Review Board (IRB) of the Albert Einstein College of Medicine. Written informed consent was obtained from all subjects or their proxies prior to participation.

### 4.2 Sequencing and alignment, variant identification, and genotype assignment

Whole exome sequencing (WES) of the cohort was performed in collaboration with the Regeneron Genetics Center (RGC) following methods previously described (Strauss et al., 2018). Briefly, high‐quality genomic DNA was fragmented and then exome‐captured using a modified version of the xGen design available from Integrated DNA Technologies (Coralville, Iowa, USA). Captured paired‐end libraries were sequenced on the Illumina HiSeq 2500 platform using v4 chemistry, achieving an average coverage of >85% of bases at 20× or greater. To limit batch effects, ELLI, their offspring, and unrelated controls were sequenced in the same batch. Upon completion of sequencing, raw data were processed through the RGC's cloud‐based pipeline that uses standard tools for mapping, alignment, and variant calling. Sequence data were mapped to the human genome reference GRCh38 using BWA‐mem (Li & Durbin, 2009). The resultant BAM files were processed and finished after initial alignment using a combination of SAMtools, Picard (Wysoker, Tibbetts, & Fennell, 2013), and GATK for sorting, duplicate marking, and small INDEL realignment. Variant calling of single‐nucleotide variants (SNVs) and intraread INDELs was performed using GATK to produce single‐sample VCF files. Following completion of cohort sequencing, a project‐level VCF (pVCF) was compiled for downstream analyses, utilizing GLnexus (Lin et al., 2018) by jointly calling genotypes across all samples in the cohort.

### 4.3 Quality control for case–control association analysis

To remove potentially false‐positive variant and genotype calls, we performed standard quality control (QC) filtering to remove variant calls in regions that are reported to have poor mapping quality, with low‐read depth, allelic imbalance, or subthreshold genotype qualities. Specifically, we removed variants with call rate <0.98, number of alleles >2, Hardy–Weinberg Equilibrium (HWE) *p* < 0.001, or Fisher's exact HWE *p* < 0.001 (Anderson et al., 2010). We then removed variants with GATK tags that are relevant to sequencing data quality: qual by depth (QD) <3, Variant Quality Score (VQSLOD) <0, or Mapping Quality Zero Read 1. After QC filtering, we obtained total of 841,702 nonredundant variants for the full cohort. We continued with 777,023 autosomal only variants with minimum allele count (MAC) of 1 that were divided into three sets for ELLI (*N* = 623,003 variants), offspring (*N* = 656,599 variants), and controls (*N* = 609,864) (Figure S1) some of which are unique to each group, or shared by 2 of 3 groups. These variant sets were used to compare against a master pathogenic dataset (comprised of HGMD variants and ClinVar variants as described below) for downstream analyses.

GRCh38 ClinVar database (downloaded April 7, 2019 from ftp://ftp.ncbi.nlm.nih.gov/pub/clinv​ar/) was filtered by clinical significance and only “likely pathogenic” or “pathogenic” annotations were retained. Variants containing conflicting evidence were removed (Landrum et al., 2017). These variants were merged according to chromosome and position with HGMD variants filtered for “High” confidence and “DM” (disease‐causing) classifications. This merged list contained 225,492 pathogenic variants. After extraction of these variants from our exome data, we obtained a dataset of 8853 pathogenic variants that was used for all analyses and will be referred to as “pathogenic variants” (Table S2).

Datasets for case–control association analysis, containing only autosomal chromosomes, were prepared for each pair of groups. Within each set of case–control pair, we performed extended sample and variant QC, according to Anderson et al. (2010). First, samples in the case–control pairs were filtered based on sample missingness (>5%), cryptic relatedness using Identity‐By‐Descent analysis (pi_hat > 0.1785) and outliers' removal using Eigensoft smartPCA. The two latter analyses were performed on a LD‐pruned subsets of variants with minor allele frequency (MAF) > 1% (Anderson et al., 2010). Since we sampled 159 direct offspring of ELLI, we wanted to check whether including them would affect the analyses; therefore, we filtered the offspring–ELLI pair for cryptic relatedness with a less stringent pi_hat (0.43), removing only First‐degree relatives. The variants in the case–control pairs were filtered by missingness (>10%), MAF > 0.1%, differential missingness between case and control (*p* < 0.00001), and departure from Hardy–Weinberg equilibrium in the control data (*p* < 0.000001). This QC resulted in 1084 samples and 459,589 variants for the control–offspring pair, 1011 samples and 454,588 variants for the control–ELLI pair, 820 samples and 483,075 variants for the offspring–ELLI pair (stringent relatedness filter), and 986 samples and 502,776 variants for the second version of the offspring–ELLI pair (looser relatedness filter). Principal Component Analysis (PCA) of all variants from case–control pairs was performed using smartPCA by Eigensoft with default settings (Price et al., 2006) in order to characterize population substructure prior to proceeding with statistical and bioinformatic analyses (Figure S3).

The final preparation for the case–control association analysis was the extraction of the pathogenic variants from our case–control pairs. This extraction resulted in 7288, 7470, and 7062 variants for ELLI, offspring, and controls, respectively. Case–control association analysis using allelic model in Plink 1.9 software (Chang et al., 2015; Marees et al., 2018) was conducted on the resulting pairs. Inflation was tested using Q‐Q plots (Clayton, 2020) revealing a slightly deflated genomic inflation factor with small variation from expected distribution (0.832–1.03) that likely resulted from the inclusion of rare variants in the analysis (MAF > 0.1%) (Figure S6). Manhattan plots were created using the R package qqman (Turner, 2014).

### 4.4 Variant annotation

Overlapping variants between each of our group variant sets and the pathogenic variants were further annotated using Variant Effect Predictor (VEP) by Ensembl (Yates et al., 2016) (release 94, https://www.ensem​bl.org/Tools/​VEP) to obtain predictions and annotations of variants within groups. Coding consequence and biotype categories were of special interest due to their possible clinical consequences.

### 4.5 Mutation load and eQTL characterization

This analysis was performed on the three groups' (ELLI, offspring, and control) data that were filtered only for autosomal variants and MAC = 1. We tested mutation load in 3 sets of our pathogenic variants data: (1) the full pathogenic variants dataset, (2) common age‐associated disease (T2D, Stroke, Cancer, CVDs and myocardial infarction, Alzheimer's, Parkinson, Dementia, and COPD) susceptibility variants, which were filtered for out of the pathogenic variants set (Table S8), and (3) a more strict filter of pathogenic variants including only pathogenic variants with at least 2 literature reports (2*) for ClinVar and only high confidence disease‐causing variants from HGMD (Table S5). The age‐associated diseases (2) analysis was performed for all diseases together and for each disease separately. The strict filtered variants' (3) analyses were performed both as a whole set and as a categorized set considering mode of inheritance (autosomal recessive, autosomal dominant, and both autosomal recessive and dominant modes). For analyses 1‐3, hetero‐ and homozygous variants were counted in each group; median and interquartile range (IQR) were calculated for the number of variants and statistically significant differences between the groups were evaluated using the nonparametric Kruskal–Wallis (KW) test. In cases of statistical significance, the analysis was followed by the nonparametric Dunn test (Daniel, 1990) (post hoc pairwise comparison), with Bonferroni correction. In order to evaluate the difference in the presence of known eQTLs between the groups, we further queried the pathogenic variants from each group in 2 large eQTL (SCAN (Zhang et al., 2015) and GRASP (Eicher et al., 2015; Leslie, O'Donnell, & Johnson, 2014)) collections. These collections are freely available and contain lists of reported eQTLs from various studies. The SCAN database was queried using Rs numbers of the variants (Rs numbers obtained from Kaviar annotation tool (Glusman, Caballero, Mauldin, Hood, & Roach, 2011)), and the GRASP was queried using chromosomal positions. This query was conducted in aim to search for known eQTL influencing variants in attempt to gain more biological insight on variants that may modulate the differences between the groups.

### 4.6 Polygenic risk score analysis

PRS was conducted using PRSice software (Euesden, Lewis, & O'Reilly, 2015) and longevity as the tested trait. Training data obtained from the CHARGE (Cohorts for Heart and Aging Research in Genomic Epidemiology) consortium, using the “90th percentile cases all controls” file available at https://www.longe​vityg​enomi​cs.org/downl​oads. For the test data, we used the case–control analyses outputs containing the full dataset containing all variants (before filtering nonpathogenic variants), as indicated in PRSice‐2 (Choi & O'Reilly, 2019) instructions. A total of 490, 493, and 503 variants were used for the analyses in controls–ELLI, control–offspring, and offspring–ELLI, respectively.

## CONFLICT OF INTEREST

PSC is a former Postdoctoral Associate of the Regeneron Genetics Center and received salary as compensation. CGJ and ARS are full‐time employees of the Regeneron Genetics Center from Regeneron Pharmaceuticals and receive salary and stock options as part of compensation. All other authors declare no conflict of interest.

## AUTHOR CONTRIBUTIONS

DG, GL, SM, NB, and GA performed the study design; DG, GL, SM, TG, RGC, JD, and GA were involved in data acquisition; DG, GL, PSC, CGJ, JD, ARS, and GA performed the analyses; DG, GL, SM, PSC, CGJ, JD, ARS, NB, and GA wrote and critically revised the manuscript.

## Data Availability

Data products from this study will be made available to researchers, collaborators and analysts without cost and upon request. Tables S2, S5, S8 and a.BIM format file available for sharing through a Google Drive link, which may be accessed via email to the corresponding author. Shared Google Drive link is required to access or download files. As part of the sharing process, users must agree to the conditions of use governing access to the public release of data, including restrictions against attempting to identify study participants, necessity of destruction of data after analyses are completed, reporting responsibilities, restrictions on redistribution of data to third parties, and proper acknowledgement of the data resource. Authorized users will receive user support as well as information related to errors in the data, notice of future releases, and publication lists. The information provided to users will not be used for commercial purposes, and will not be redistributed to third parties.

## References

[acel13216-bib-0001] Andersen, S. L. , Sebastiani, P. , Dworkis, D. A. , Feldman, L. , & Perls, T. T. (2012). Health span approximates life span among many supercentenarians: Compression of morbidity at the approximate limit of life span. The Journals of Gerontology: Series A, 67A(4), 395–405. 10.1093/gerona/glr223 PMC3309876 22219514

[acel13216-bib-0002] Anderson, C. A. , Pettersson, F. H. , Clarke, G. M. , Cardon, L. R. , Morris, A. P. , & Zondervan, K. T. (2010). Data quality control in genetic case‐control association studies. Nature Protocols, 5, 1564. 21085122 10.1038/nprot.2010.116 PMC3025522

[acel13216-bib-0003] Atzmon, G. , Cho, M. , Cawthon, R. M. , Budagov, T. , Katz, M. , Yang, X. , … Suh, Y. (2010). Evolution in health and medicine Sackler colloquium: Genetic variation in human telomerase is associated with telomere length in Ashkenazi centenarians. Proceedings of the National Academy of Sciences of the United States of America, 107(Suppl), 1710–1717. 10.1073/pnas.0906191106 19915151 PMC2868292

[acel13216-bib-0004] Balistreri, C. R. , Candore, G. , Accardi, G. , Buffa, S. , Bulati, M. , Martorana, A. , … Caruso, C. (2014). Centenarian offspring: A model for understanding longevity. Current Vascular Pharmacology, 12(5), 718–725. 10.2174/1570161111666131219113544 24350924

[acel13216-bib-0005] Barzilai, N. , Gabriely, I. , Gabriely, M. , Iankowitz, N. , & Sorkin, J. D. (2001). Offspring of centenarians have a favorable lipid profile. Journal of the American Geriatrics Society, 49, 76–79. 11207846 10.1046/j.1532-5415.2001.49013.x

[acel13216-bib-0006] Beekman, M. , Blanché, H. , Perola, M. , Hervonen, A. , Bezrukov, V. , Sikora, E. , … Franceschi, C. (2013). Genome‐wide linkage analysis for human longevity: Genetics of healthy aging study. Aging Cell, 12, 184–193. 23286790 10.1111/acel.12039 PMC3725963

[acel13216-bib-0007] Bergman, A. , Atzmon, G. , Ye, K. , MacCarthy, T. , & Barzilai, N. (2007). Buffering mechanisms in aging: A systems approach toward uncovering the genetic component of aging. PLoS Computational Biology, 3, e170 10.1371/journal.pcbi.0030170 17784782 PMC1963511

[acel13216-bib-0008] Bick, A. G. , Flannick, J. , Ito, K. , Cheng, S. , Vasan, R. S. , Parfenov, M. G. , … Seidman, C. (2012). Burden of rare sarcomere gene variants in the Framingham and Jackson Heart Study cohorts. American Journal of Human Genetics, 91, 513–519. 22958901 10.1016/j.ajhg.2012.07.017 PMC3511985

[acel13216-bib-0009] Broer, L. , Buchman, A. S. , Deelen, J. , Evans, D. S. , Faul, J. D. , Lunetta, K. L. , … Tanaka, T. (2014). GWAS of longevity in CHARGE consortium confirms APOE and FOXO3 candidacy. Journals of Gerontology Series A: Biomedical Sciences and Medical Sciences, 70, 110–118. 10.1093/gerona/glu166 PMC4296168 25199915

[acel13216-bib-0010] Brooks‐Wilson, A. R. (2013). Genetics of healthy aging and longevity. Human Genetics, 132, 1323–1338. 23925498 10.1007/s00439-013-1342-z PMC3898394

[acel13216-bib-0011] Carmi, S. , Hui, K. Y. , Kochav, E. , Liu, X. , Xue, J. , Grady, F. , … Pe'er, I. (2014). Sequencing an Ashkenazi reference panel supports population‐targeted personal genomics and illuminates Jewish and European origins. Nature Communications, 5(1), 1–9. 10.1038/ncomms5835 PMC4164776 25203624

[acel13216-bib-0012] Chang, C. C. , Chow, C. C. , Tellier, L. C. A. M. , Vattikuti, S. , Purcell, S. M. , & Lee, J. J. (2015). Second‐generation PLINK: Rising to the challenge of larger and richer datasets. Gigascience, 4, 7. 25722852 10.1186/s13742-015-0047-8 PMC4342193

[acel13216-bib-0013] Choi, S. W. , & O'Reilly, P. F. (2019). PRSice‐2: Polygenic risk score software for biobank‐scale data. GigaScience, 8(7), 1–6. 10.1093/gigascience/giz082 PMC6629542 31307061

[acel13216-bib-0014] Clayton, D. (2020). snpStats: SnpMatrix and XSnpMatrix classes and methods. R package version 1.38.0.

[acel13216-bib-0015] Daniel, W. W. (ed) (1990). Multiple Comparisons Applied nonparametric statistics. The Duxbury Advanced Series in Statistics and Decision Sciences, 2nd ed., (pp. 240–244). University of Michigan, Boston, MA, USA: PWS‐KENT Pub.

[acel13216-bib-0016] Dapeng, H. , Wang, L. , & Di, L. (2016). Distinct mutation accumulation rates among tissues determine the variation in cancer risk. Scientific Reports, 6, 19458. 26785814 10.1038/srep19458 PMC4726417

[acel13216-bib-0017] Deelen, J. , Beekman, M. , Uh, H.‐W. , Helmer, Q. , Kuningas, M. , Christiansen, L. , … Slagboom, P. E. (2011). Genome‐wide association study identifies a single major locus contributing to survival into old age; the APOE locus revisited. Aging Cell, 10, 686–698. 21418511 10.1111/j.1474-9726.2011.00705.x PMC3193372

[acel13216-bib-0018] Eicher, J. D. , Landowski, C. , Stackhouse, B. , Sloan, A. , Chen, W. , Jensen, N. , … Johnson, A. D. (2015). GRASP v2.0: An update on the Genome‐wide repository of associations between SNPs and phenotypes. Nucleic Acids Research, 43, D799–D804. 10.1093/nar/gku1202 25428361 PMC4383982

[acel13216-bib-0019] Erikson, G. A. , Bodian, D. L. , Rueda, M. , Molparia, B. , Scott, E. R. , Scott‐Van Zeeland, A. A. , … Torkamani, A. (2016). Whole‐genome sequencing of a healthy aging cohort. Cell, 165, 1002–1011. 27114037 10.1016/j.cell.2016.03.022 PMC4860090

[acel13216-bib-0020] Euesden, J. , Lewis, C. M. , & O'Reilly, P. F. (2015). PRSice: Polygenic risk score software. Bioinformatics, 31(9), 1466–1468. 10.1093/bioinformatics/btu848 25550326 PMC4410663

[acel13216-bib-0021] Freudenberg‐Hua, Y. , Freudenberg, J. , Vacic, V. , Abhyankar, A. , Emde, A.‐K. , Ben‐Avraham, D. , … Davies, P. (2014). Disease variants in genomes of 44 centenarians. Molecular Genetics & Genomic Medicine, 2, 438–450. 25333069 10.1002/mgg3.86 PMC4190879

[acel13216-bib-0022] Freudenberg‐Hua, Y. , Li, W. , Abhyankar, A. , Vacic, V. , Cortes, V. , Ben‐Avraham, D. , … Consortium, T.‐ D.‐G. (2016). Differential burden of rare protein truncating variants in Alzheimer's disease patients compared to centenarians. Human Molecular Genetics, 25, 3096–3105. 27260402 10.1093/hmg/ddw150 PMC5181592

[acel13216-bib-0023] Glusman, G. , Caballero, J. , Mauldin, D. E. , Hood, L. , & Roach, J. C. (2011). Kaviar: An accessible system for testing SNV novelty. Bioinformatics, 27, 3216–3217. 10.1093/bioinformatics/btr540 21965822 PMC3208392

[acel13216-bib-0024] Gubbi, S. , Schwartz, E. , Crandall, J. , Verghese, J. , Holtzer, R. , Atzmon, G. , … Milman, S. (2017). Effect of exceptional parental longevity and lifestyle factors on prevalence of cardiovascular disease in offspring. The American Journal of Cardiology, 120, 2170–2175. 10.1016/j.amjcard.2017.08.040 29050682 PMC5698168

[acel13216-bib-0025] Gutman, D. , Rivkin, E. , Fadida, A. , Sharvit, L. , Hermush, V. , Rubin, E. , … Atzmon, G. (2020). Exceptionally long‐lived individuals (ELLI) demonstrate slower aging rate calculated by DNA methylation clocks as possible modulators for healthy longevity. International Journal of Molecular Sciences, 21(2), 615. 10.3390/ijms21020615 PMC7013521 31963520

[acel13216-bib-0026] Holstege, H. , Pfeiffer, W. , Sie, D. , Hulsman, M. , Nicholas, T. J. , Lee, C. C. , … Sistermans, E. A. (2014). Somatic mutations found in the healthy blood compartment of a 115‐yr‐old woman demonstrate oligoclonal hematopoiesis. Genome Research, 24, 733–742. 24760347 10.1101/gr.162131.113 PMC4009603

[acel13216-bib-0027] Ismail, K. , Nussbaum, L. , Sebastiani, P. , Andersen, S. , Perls, T. , Barzilai, N. , & Milman, S. (2016). Compression of morbidity is observed across cohorts with exceptional longevity. Journal of the American Geriatrics Society, 64(8), 1583–1591. 10.1111/jgs.14222 27377170 PMC4988893

[acel13216-bib-0028] Joshi, P. K. , Fischer, K. , Schraut, K. E. , Campbell, H. , Esko, T. , & Wilson, J. F. (2016). Variants near CHRNA3/5 and APOE have age‐and sex‐related effects on human lifespan. Nature Communications, 7, 11174. 10.1038/ncomms11174 PMC5438072 27029810

[acel13216-bib-0029] Landrum, M. J. , Lee, J. M. , Benson, M. , Brown, G. R. , Chao, C. , Chitipiralla, S. , … Jang, W. (2017). ClinVar: improving access to variant interpretations and supporting evidence. Nucleic Acids Research, 46, D1062–D1067. 10.1093/nar/gkx1153 PMC5753237 29165669

[acel13216-bib-0030] Lencz, T. , Yu, J. , Palmer, C. , Carmi, S. , Ben‐Avraham, D. , Barzilai, N. , … Pe’er, I. (2018). High‐depth whole genome sequencing of an Ashkenazi Jewish reference panel: enhancing sensitivity, accuracy, and imputation. Human Genetics, 137, 343–355, 10.1007/s00439-018-1886-z 29705978 PMC6954822

[acel13216-bib-0031] Leslie, R. , O'Donnell, C. J. , & Johnson, A. D. (2014). GRASP: Analysis of genotype‐phenotype results from 1390 Genome‐wide association studies and corresponding open access database. Bioinformatics, 30, i185–i194. 10.1093/bioinformatics/btu273 24931982 PMC4072913

[acel13216-bib-0032] Li, H. , & Durbin, R. (2009). Fast and accurate short read alignment with Burrows‐Wheeler transform. Bioinformatics, 25, 1754–1760. 19451168 10.1093/bioinformatics/btp324 PMC2705234

[acel13216-bib-0033] Lin, M. F. , Rodeh, O. , Penn, J. , Bai, X. , Krasheninina, O. , Salerno, W. J. , & Reid, J. G. (2018). Glnexus: Joint variant calling for large cohort sequencing. bioRxiv, 343970.

[acel13216-bib-0034] Lodato, M. A. & Walsh, C. A. (2019). Genome aging: somatic mutation in the brain links age‐related decline with disease and nominates pathogenic mechanisms. Human Molecular Genetics, 28(R2), R197–R206. 31578549 10.1093/hmg/ddz191 PMC6872434

[acel13216-bib-0035] Marees, A. T. , de Kluvier, H. , Stringer, S. , Vorspan, F. , Curis, E. , Marie‐Claire, C. , & Derks, E. M. (2018). A tutorial on conducting Genome‐wide association studies: Quality control and statistical analysis. International Journal of Methods in Psychiatric Research, 27(2), e1608 10.1002/mpr.1608 29484742 PMC6001694

[acel13216-bib-0036] Milholland, B. , Auton, A. , Suh, Y. , & Vijg, J. (2015). age‐related somatic mutations in the cancer genome. Oncotarget, 6(28), 24627–24635. 26384365 10.18632/oncotarget.5685 PMC4694783

[acel13216-bib-0037] Milman, S. , & Barzilai, N. (2015). Dissecting the Mechanisms Underlying Unusually Successful Human Health Span and Life Span. Cold Spring Harbor Perspectives in Medicine, 6, a025098 10.1101/cshperspect.a025098 26637439 PMC4691799

[acel13216-bib-0038] Nebel, A. , & Schreiber, S. (2004). GEHA–the pan‐European "Genetics of Healthy Aging" project. Science of Aging Knowledge Environment: SAGE KE, 2004, pe23 10.1126/sageke.2004.21.pe23 15163849

[acel13216-bib-0039] Park, J. S. , Lee, J. , Jung, E. S. , Kim, M. , Kim, I. B. , Son, H. , … Lee, J. H. (2019). Brain somatic mutations observed in Alzheimer's disease associated with aging and dysregulation of tau phosphorylation. Nature Communications, 10, 3090. 10.1038/s41467-019-11000-7 PMC6626023 31300647

[acel13216-bib-0040] Patel, T. , Brooks, K. J. , Turton, J. , Chaudhury, S. , Guetta‐Baranes, T. , Guerreiro, R. , … Morgan, K. (2017). Whole‐exome sequencing of the BDR cohort: evidence to support the role of the PILRA gene in Alzheimer's disease. Neuropathology and Applied Neurobiology, 44(5), 506–521. 10.1111/nan.12452 PMC6005734 29181857

[acel13216-bib-0041] Pilling, L. C. , Atkins, J. L. , Bowman, K. , Jones, S. E. , Tyrrell, J. , Beaumont, R. N. , … Melzer, D. (2016). Human longevity is influenced by many genetic variants: evidence from 75,000 UK Biobank participants. Aging, 8, 547–560. 10.18632/aging.100930 27015805 PMC4833145

[acel13216-bib-0042] Price, A. L. , Patterson, N. J. , Plenge, R. M. , Weinblatt, M. E. , Shadick, N. A. , & Reich, D. (2006). Principal components analysis corrects for stratification in Genome‐wide association studies. Nature Genetics, 38, 904. 16862161 10.1038/ng1847

[acel13216-bib-0043] Puca, A. A. , Spinelli, C. , Accardi, G. , Villa, F. , & Caruso, C. (2018). Centenarians as a model to discover genetic and epigenetic signatures of healthy ageing. Mechanisms of Ageing and Development, 174, 95–102. 29096878 10.1016/j.mad.2017.10.004

[acel13216-bib-0044] Sebastiani, P. , Nussbaum, L. , Andersen, S. L. , Black, M. J. , & Perls, T. T. (2015). Increasing sibling relative risk of survival to older and older ages and the importance of precise definitions of "aging", "life span", and "longevity". The Journals of Gerontology: Series A, 71(3), 340–346. 10.1093/gerona/glv020 PMC4757962 25814633

[acel13216-bib-0045] Sebastiani, P. , Solovieff, N. , DeWan, A. T. , Walsh, K. M. , Puca, A. , Hartley, S. W. , … Perls, T. T. (2012). Genetic signatures of exceptional longevity in humans. PLoS One, 7(1), e29848. 22279548 10.1371/journal.pone.0029848 PMC3261167

[acel13216-bib-0046] Sebastiani, P. , Sun, F. X. , Andersen, S. L. , Lee, J. H. , Wojczynski, M. K. , Sanders, J. L. , … Perls, T. T. (2013). Families enriched for exceptional longevity also have increased health‐span: Findings from the long life family study. Frontiers in Public Health, (1). 10.3389/fpubh.2013.00038 PMC3859985 24350207

[acel13216-bib-0047] Stenson, P. D. , Mort, M. , Ball, E. V. , Evans, K. , Hayden, M. , Heywood, S. , … Cooper, D. N. (2017). The Human Gene Mutation Database: towards a comprehensive repository of inherited mutation data for medical research, genetic diagnosis and next‐generation sequencing studies. Human Genetics, 136, 665–677. 10.1007/s00439-017-1779-6 28349240 PMC5429360

[acel13216-bib-0048] Stevenson, M. , Bae, H. , Schupf, N. , Andersen, S. , Zhang, Q. , Perls, T. , & Sebastiani, P. (2015). Burden of disease variants in participants of the Long Life Family Study. Aging, 7, 123–132. 10.18632/aging.100724 25664523 PMC4359694

[acel13216-bib-0049] Strauss, K. A. , Gonzaga‐Jauregui, C. , Brigatti, K. W. , Williams, K. B. , King, A. K. , Van Hout, C. , … Puffenberger, E. G. (2018). Genomic diagnostics within a medically underserved population: Efficacy and implications. Genetics in Medicine, 20, 31. 28726809 10.1038/gim.2017.76

[acel13216-bib-0050] Teixeira, L. , Araújo, L. , Jopp, D. , & Ribeiro, O. (2017). Centenarians in Europe. Maturitas, 104, 90–95. 28923181 10.1016/j.maturitas.2017.08.005

[acel13216-bib-0051] Tesi, N. , der Lee, S. J. , Hulsman, M. , Jansen, I. E. , Stringa, N. , van Schoor, N. , … Reinders, M. J. T. (2018). Centenarian controls increase variant effect sizes by an average twofold in an extreme case–extreme control analysis of Alzheimer's disease. European Journal of Human Genetics, 27, 244–253. 30258121 10.1038/s41431-018-0273-5 PMC6336855

[acel13216-bib-0052] Tindale, L. C. , Zeng, A. , Bretherick, K. L. , Leach, S. , Thiessen, N. , & Brooks‐Wilson, A. R. (2015). Burden of common complex disease variants in the Exomes of two healthy centenarian brothers. Gerontology, 62, 58–62. 10.1159/000430462 26066993

[acel13216-bib-0053] Turner, S. D. (2014). qqman: An R package for visualizing GWAS results using QQ and manhattan plots. BioRxiv.

[acel13216-bib-0054] Wysoker, A. , Tibbetts, K. , & Fennell, T. (2013). Picard tools version 1.96. Retrieved from http://picard.sourc​eforge.net. 10.2307/41303121.

[acel13216-bib-0055] Yates, A. , Akanni, W. , Amode, M. R. , Barrell, D. , Billis, K. , Carvalho‐Silva, D. , … Flicek, P. (2016). Ensembl 2016. Nucleic Acids Research, 44, D710–716. 10.1093/nar/gkv1157 [doi] 26687719 PMC4702834

[acel13216-bib-0056] Ye, K. , Beekman, M. , Lameijer, E.‐W. , Zhang, Y. , Moed, M. H. , van den Akker, E. B. , … Slagboom, P. E. (2013). Aging as accelerated accumulation of somatic variants: Whole‐genome sequencing of centenarian and middle‐aged monozygotic twin pairs. Twin Research and Human Genetics, 16, 1026–1032. 24182360 10.1017/thg.2013.73

[acel13216-bib-0057] Zhang, W. , Gamazon, E. R. , Zhang, X. , Konkashbaev, A. , Liu, C. , Szilagyi, K. L. , … Cox, N. J. (2015). SCAN database: Facilitating integrative analyses of cytosine modification and expression QTL. Database: The Journal of Biological Databases and Curation, 2015, bav025 10.1093/database/bav025 25818895 PMC4375357

