# Supplementary material for: Similar burden of pathogenic coding variants in exceptionally long‐lived individuals and individuals without exceptional longevity
Source: Aging Cell. 2020 Aug 29;19(10):e13216. doi: 10.1111/acel.13216 (PMC7576295; doi:10.1111/acel.13216)
Supplement: Supplementary file 1 [file ACEL-19-e13216-s001.docx]

**Supporting Information**

**
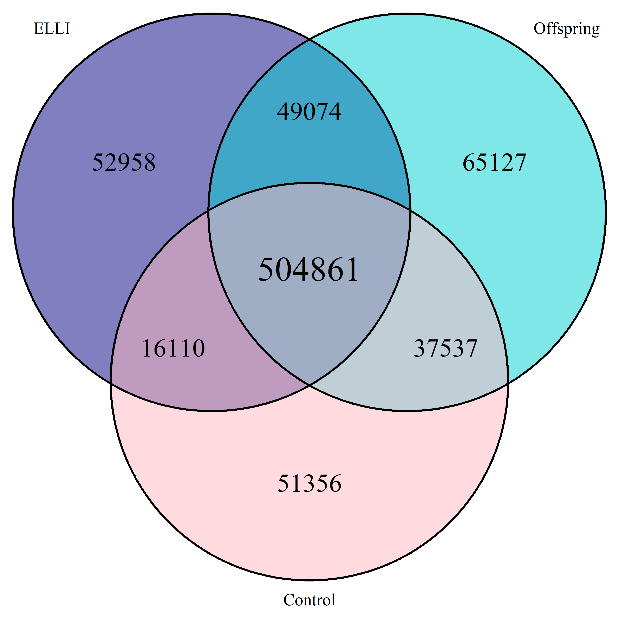
**

**Supporting Figure** **1: Total variants.** Venn diagram showing the number of all variants present in each group and in the unions between them.

777,023 autosomal variants

515 ELLI

832 Offspring

532 Controls

VEP annotation, mutation load, and eQTL analyses

Case-control association tests

Pathogenic variant subsetting

* Missing > 5%

* Relatedness

* Outliers

* HWE

MAC = 1

**Supporting Figure** **2: Schematic view of pipeline analysis**


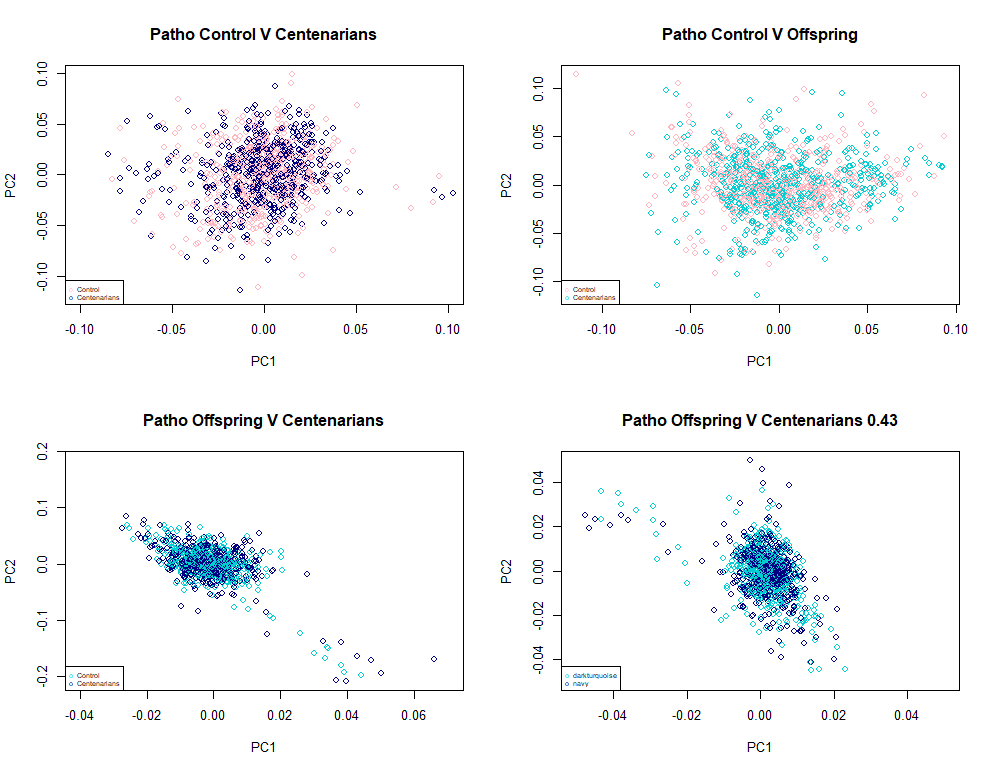

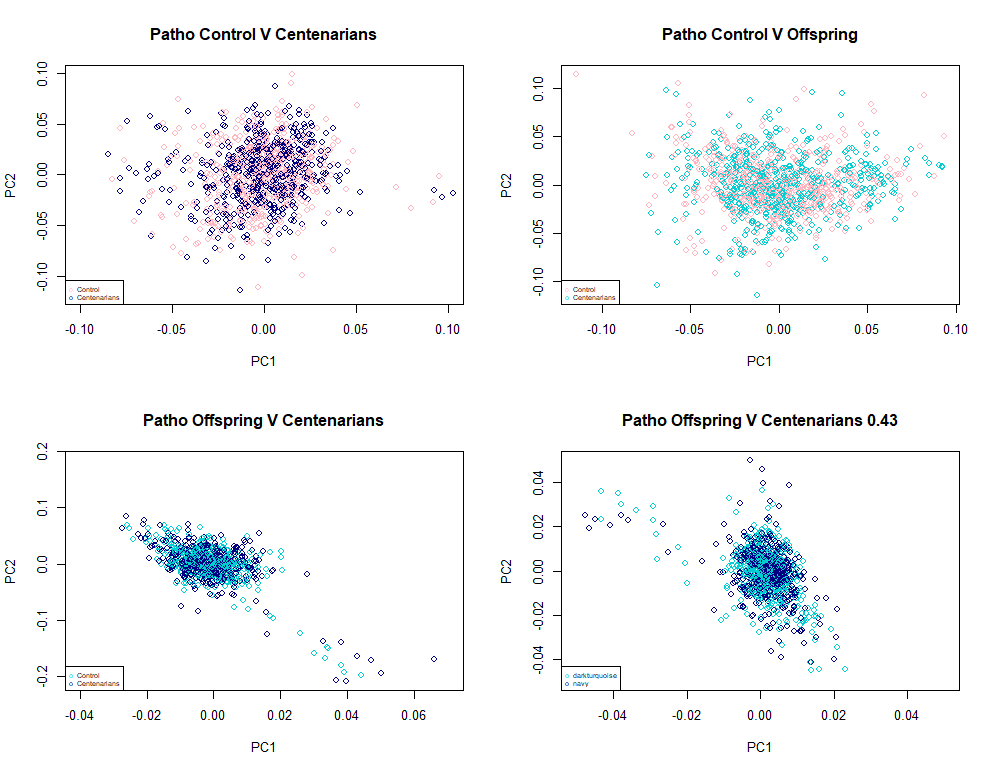

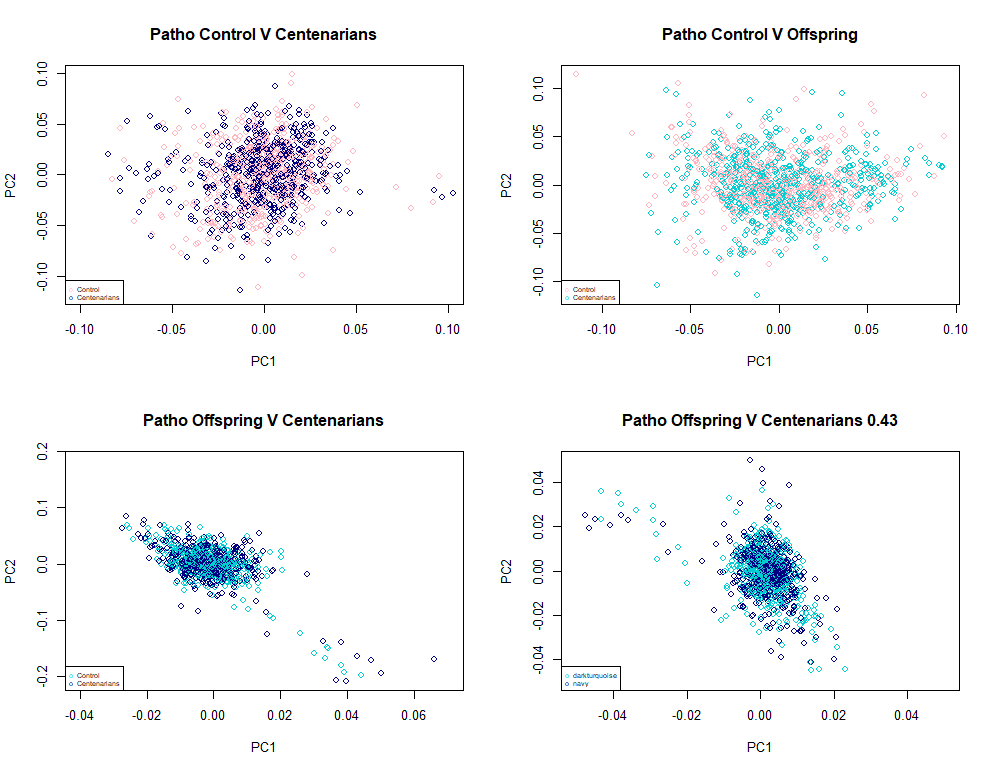


**A**

**B**

**C**

**Supporting Figure** **3: Principal component analysis** of three case-control analyses of all variants (following QC and filtering). **A.** Control vs. ELLI, **B.** Control vs. Offspring, and **C.** Offspring vs. ELLI without removing subjects according to IBD.

**Supporting Figure** **4: VEP summary of general and coding consequences** of pathogenic variants in each group determined from exome sequencing. Outer ring presents the findings in the ELLI group, middle ring represents the findings in the offspring, and the inner ring represents the findings in the control group. Numbers represent the percent of variants that fall into each consequence prediction category.

**Supporting Figure** **5: Frequency of biotypes** of variants in each group, as determined by Ensembl VEP.


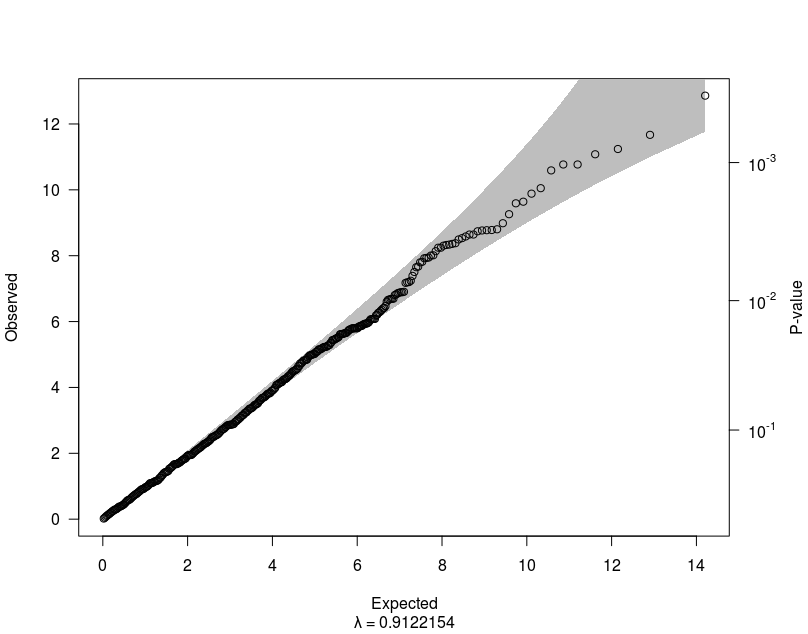


**A**


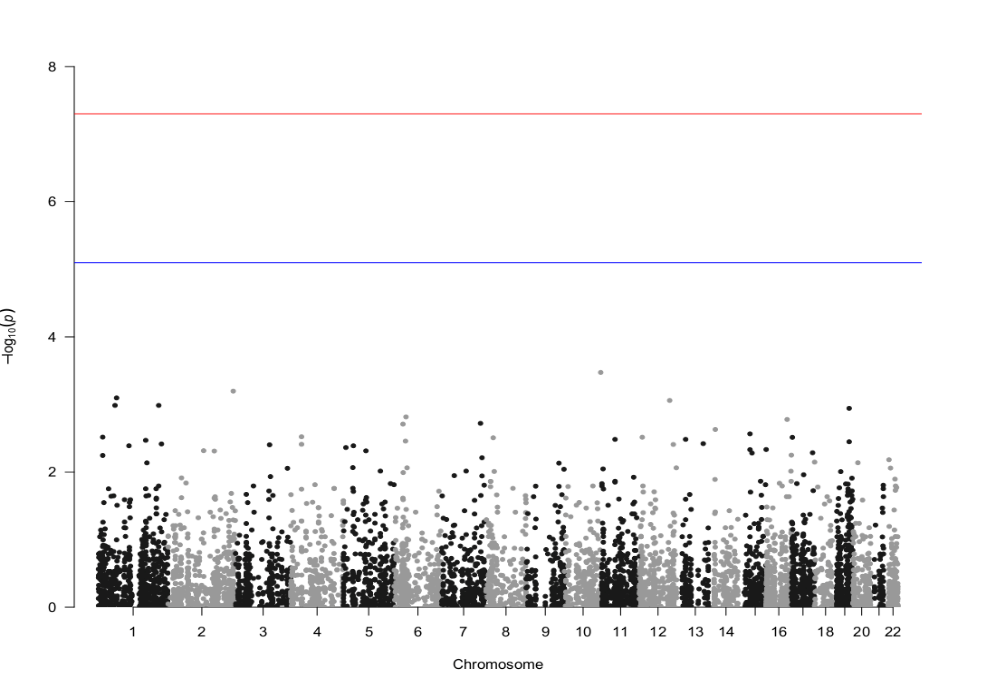


**B**

**Supporting Figure** **6: Control-ELLI case-control analysis. A.**Quartile-Quartile plot, and **B.** Manhattan plot.


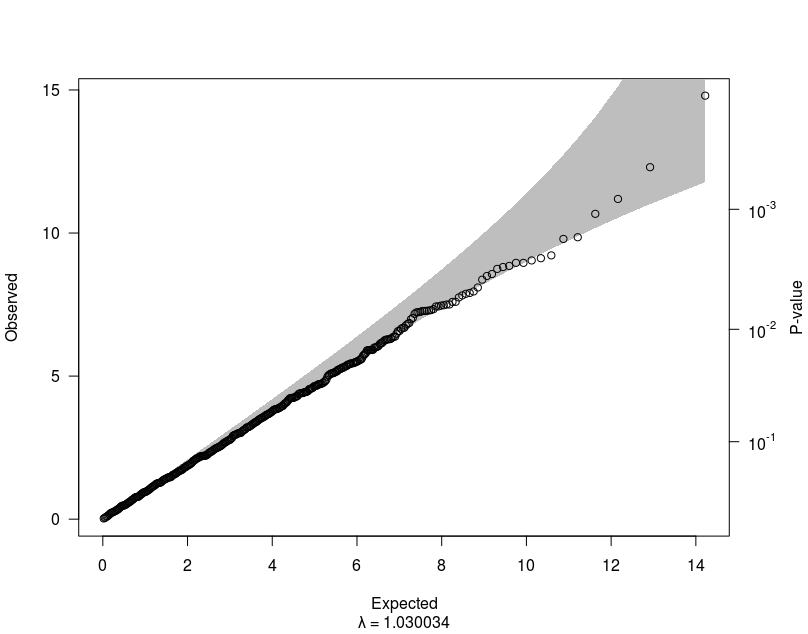

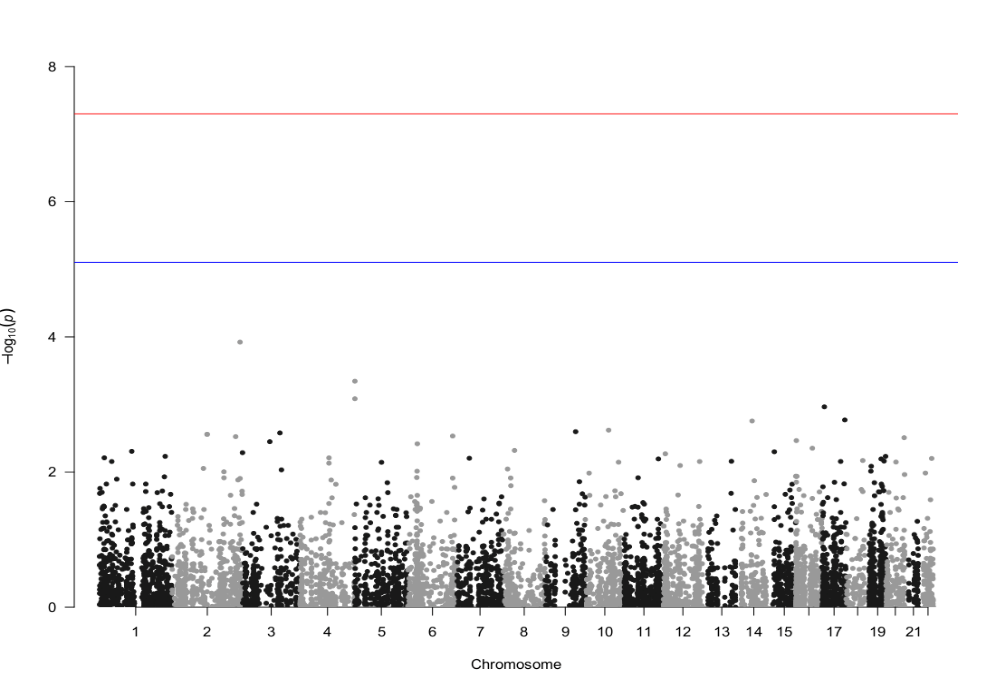


**A**

**B**

**Supporting Figure** **7: Control-offspring case-control analysis. A.**Quartile-Quartile plot, and **B.** Manhattan plot.


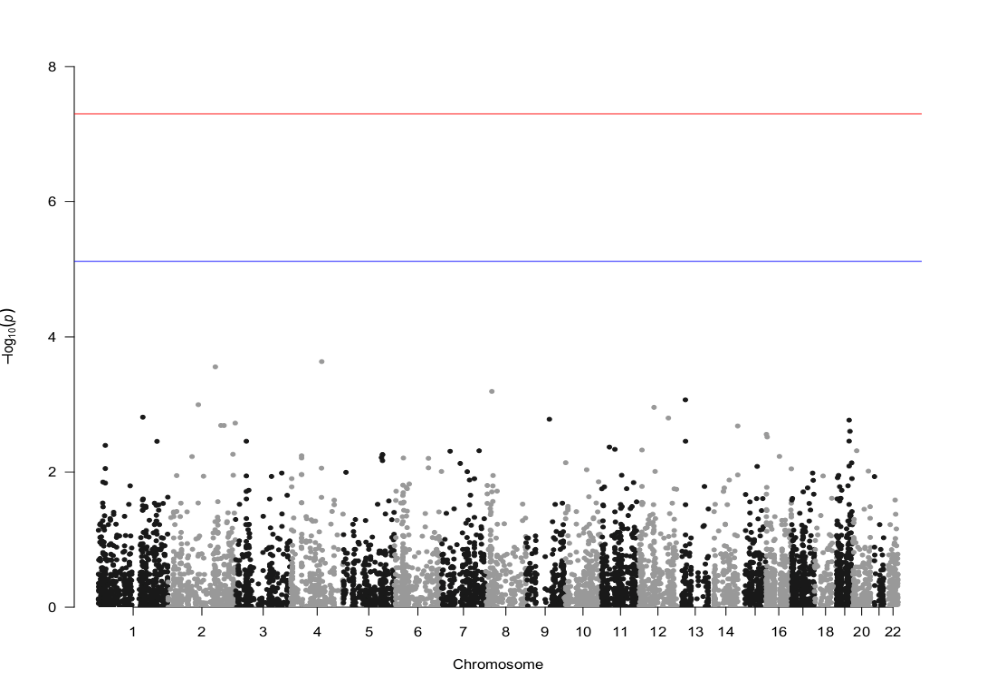

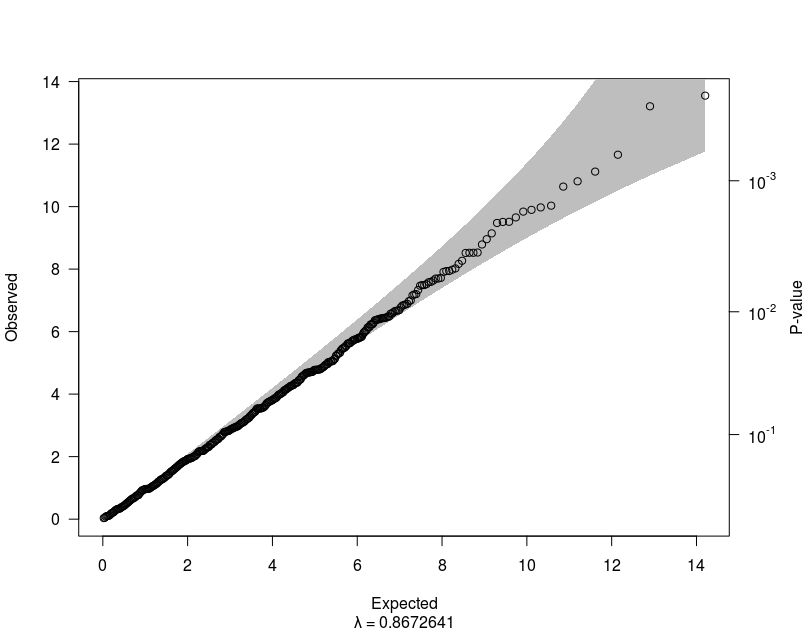


**A**

**B**

**Supporting Figure** **8: Offspring-ELLI case-control analysis. A.**Quartile-Quartile plot, and **B.** Manhattan plot.


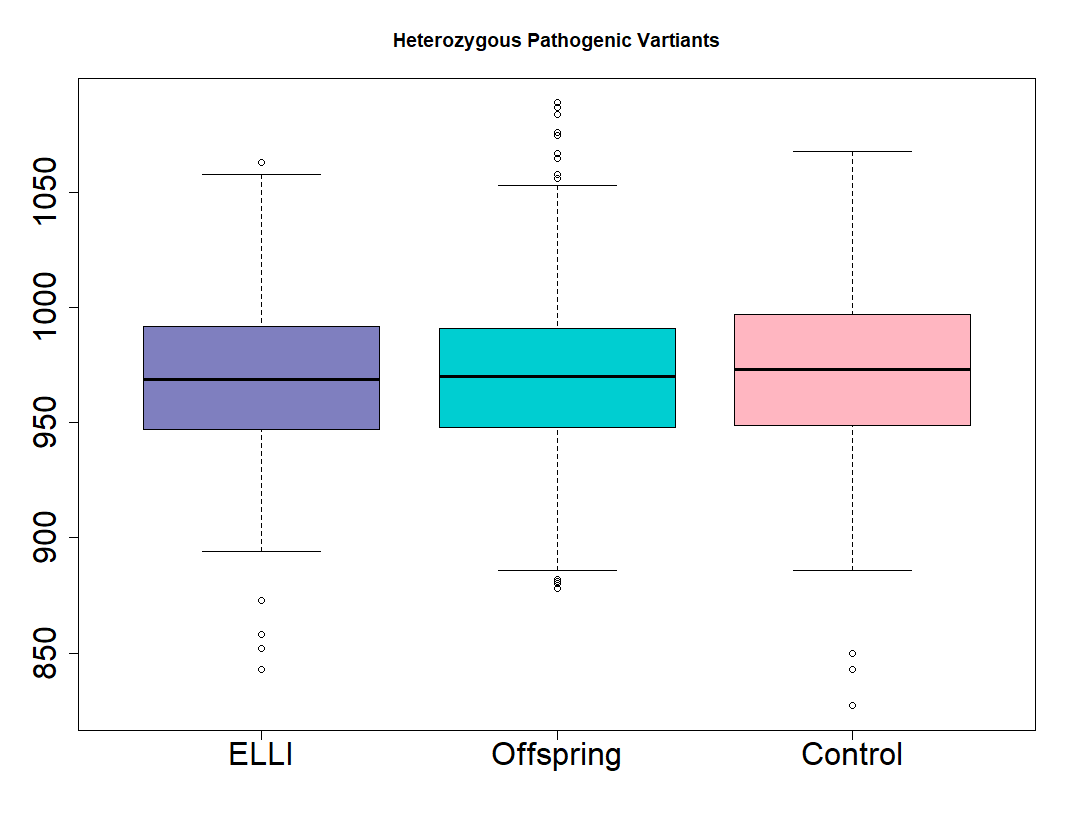

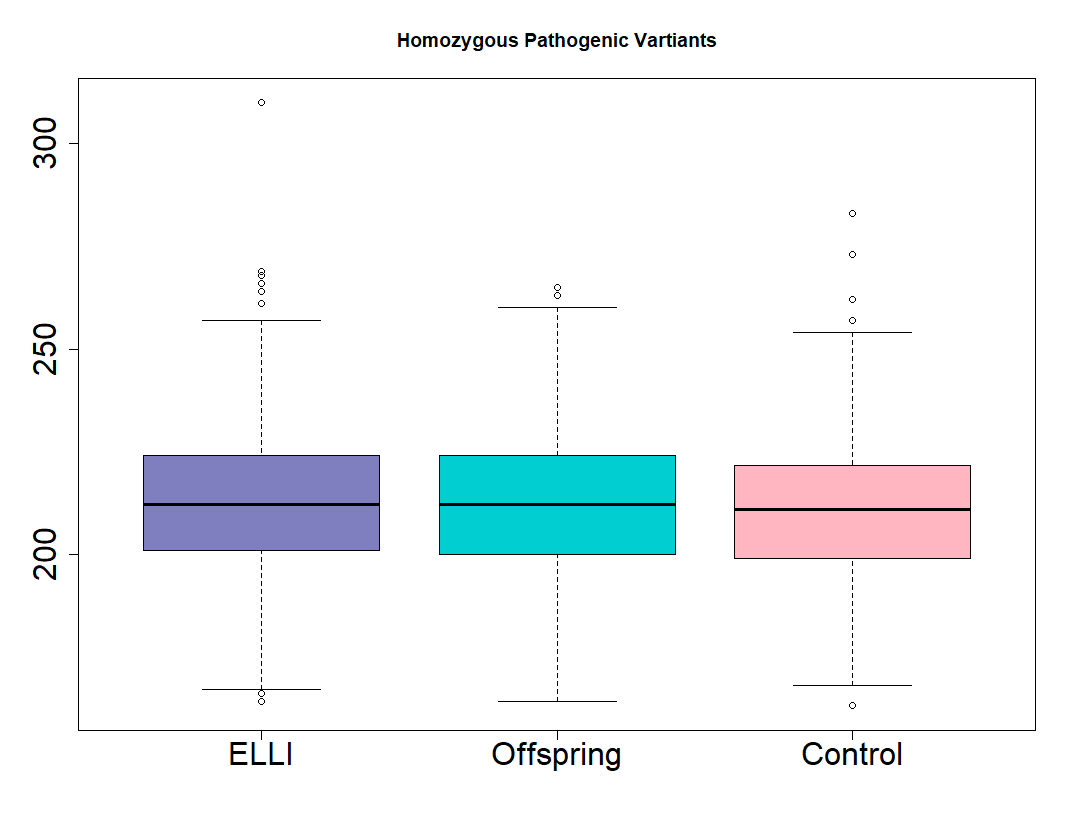


**A**

**B**

**Supporting Figure** **9: Comparison of pathogenic variants in the 3 groups.** The bold horizontal line in each box represents the median value of individual pathogenic variants in the respective distribution. The area between the top and bottom lines is the IQR. **A.** Heterozygous pathogenic variants per individual by group. **B.** Homozygous pathogenic variants per individual by group.


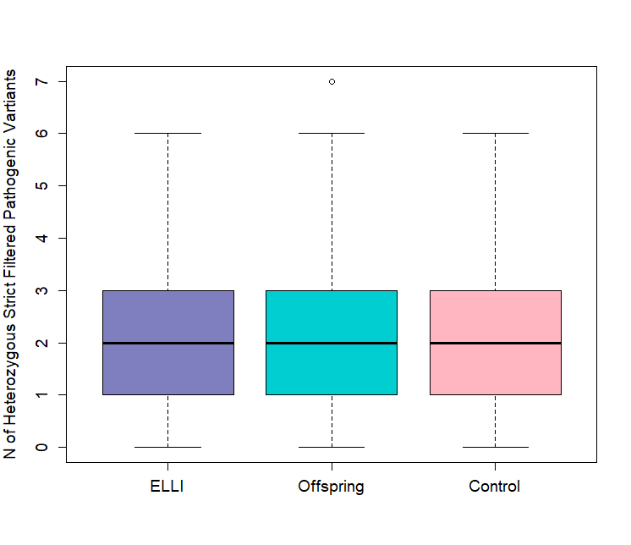

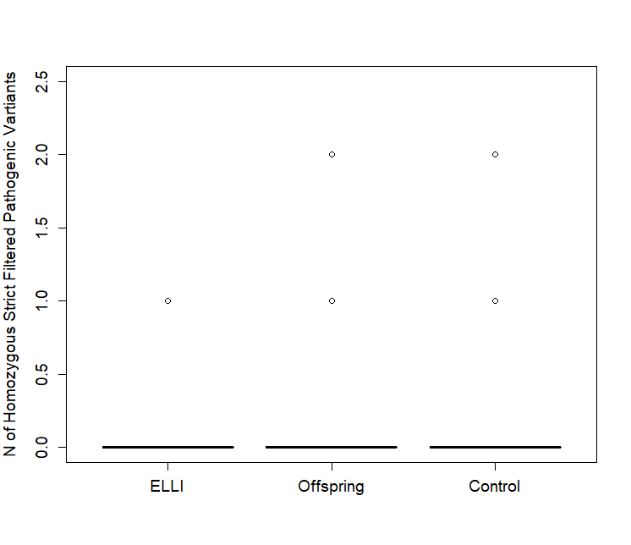


**A**

**B**

**Supporting Figure** **10: Pathogenic 2* ClinVar variants and HGMD high confidence disease-causing variants in each group.** The bold horizontal line in each box represents the median value of individual variants in the respective distribution. The area between the top and bottom lines is the IQR. **A.** Heterozygous variants per individual by group. **B.** Homozygous variants per individual by group.

**Supporting Table 3:** Pathogenic and age-associated disease related variants statistical analysis.

|  | Genotype | Group | Mean | SE | Median | IQR (Q3 – Q1) | Kruskal-­‐Wallis test, P-value | Dunn Test , Bonferroni adjusted 2-tailed p-value |
| --- | --- | --- | --- | --- | --- | --- | --- | --- |
| Pathogenic Variants | **Heterozygous** | Control | 971.78 | 1.51 | 973 | 48 | 0.2122 | - |
|  |  | Offspring | 970.24 | 1.19 | 970 | 43 |  | - |
|  |  | ELLI | 968.11 | 1.44 | 969 | 45 |  | - |
|  | **Homozygous** | Control | 211.20 | 0.77 | 211 | 22.2 | 0.2942 | - |
|  |  | Offspring | 212.51 | 0.62 | 212 | 24 |  | - |
|  |  | ELLI | 212.72 | 0.82 | 212 | 23 |  | - |
| Pathogenic Age-Associated-Disease-Related Variants subset | **Heterozygous** | Control | 159.60 | 0.50 | 160 | 16 | 0.009371 | ELLI – Ctrl: 0.0046* |
|  |  | Offspring | 159.15 | 0.40 | 159 | 14 |  | ELLI – Off: 0.0331 |
|  |  | ELLI | 157.72 | 0.50 | 157 | 15 |  | Ctrl – Off: 0.4889 |
|  | **Homozygous** | Control | 35.87 | 0.27 | 36 | 9 | 0.07456 | - |
|  |  | Offspring | 36.48 | 0.22 | 36 | 9 |  | - |
|  |  | ELLI | 36.87 | 0.28 | 37 | 8.5 |  | - |
| ClinVar Pathogenic 2* Only and HGMD High Confidence Disease Causing Only | **Heterozygous** | Control | 1.91 | 1.33 | 2 | 2 | 0.7873 |  |
|  |  | Offspring | 1.86 | 1.31 | 2 | 2 |  |  |
|  |  | ELLI | 1.91 | 1.35 | 2 | 2 |  |  |
|  | **Homozygous** | Control | 0.03 | 0.19 | 0 | 0 | 0.33 |  |
|  |  | Offspring | 0.03 | 0.17 | 0 | 0 |  |  |
|  |  | ELLI | 0.2 | 0.13 | 0 | 0 |  |  |

**Supporting Table** **1: Top 10 variants in case-control paired analyses.** All gene annotations and disease association from HGMD.

| SNP | P | OR | group pair | Gene | | Disease |
| --- | --- | --- | --- | --- | --- | --- |
| **10:129708019:C:T** | 3.36E-04 | 1.599 | Control-ELLI | | MGMT | Endometrial Cancer Risk |
| **2:233729157:T:C** | 6.34E-04 | 1.356 | Control-ELLI | | UGT1A3 | Altered Enzyme Activity |
| **1:65570758:A:G** | 8.01E-04 | 1.521 | Control-ELLI | | LEPR | Increased Birth Weight |
| **12:108224853:C:T** | 8.72E-04 | 1.414 | Control-ELLI | | WSCD2 | Diabetes Type 2 |
| **1:59926822:C:A** | 1.03E-03 | 1.952 | Control-ELLI | | CYP2J2 | Coronary Artery Disease |
| **1:216046516:C:A** | 1.03E-03 | 0.07382 | Control-ELLI | | USH2A | Retinitis Pigmentosa |
| **19:44908684:T:C** | 1.14E-03 | 0.5965 | Control-ELLI | | APOE | Apolipoprotein E Deficiency |
| **6:42965726:G:A** | 1.53E-03 | 0.5068 | Control-ELLI | | PEX6 | Zellweger Syndrome |
| **16:74740049:G:A** | 1.67E-03 | 12.7 | Control-ELLI | | FA2H | Autism Spectrum Disorder |
| **7:138722001:C:A** | 1.90E-03 | 0.3045 | Control-ELLI | | ATP6V0A4 | Distal Renal Tubular Acidosis |
| **4:110623148:C:T** | 2.32E-04 | 7.432 | Offspring-ELLI | | PITX2 | Axenfeld-Rieger Syndrome |
| **2:169637353:A:G** | 2.78E-04 | 0.6458 | Offspring-ELLI | | PPIG | Leukaemia |
| **8:23191779:C:T** | 6.40E-04 | 1.717 | Offspring-ELLI | | TNFRSF10A | Lung Cancer |
| **13:31777398:A:C** | 8.53E-04 | 3.552 | Offspring-ELLI | | RXFP2 | Cryptorchidism |
| **2:108897145:A:G** | 1.01E-03 | 3.201 | Offspring-ELLI | | EDAR | Increased Hair Thickness in Asians |
| **12:52676305:T:G** | 1.10E-03 | 4.248 | Offspring-ELLI | | KRT1 | Epidermolytic Hyperkeratosis |
| **1:159302270:T:C** | 1.54E-03 | 1.596 | Offspring-ELLI | | FCER1A | Atopic Dermatitis |
| **12:103938446:C:T** | 1.59E-03 | 7.921 | Offspring-ELLI | | HSP90B1 | Decreased Circulating IGF-1 Levels |
| **9:84002350:A:G** | 1.66E-03 | 1.413 | Offspring-ELLI | | RMI1 | Leukaemia/Myelodysplastic Syndrome/Melanoma |
| **19:44908684:T:C** | 1.71E-03 | 0.5506 | Offspring-ELLI | | APOE | Apolipoprotein E Deficiency |
| **16:47515562:G:T** | 1.43E-04 | 0.4346 | Offspring-ELLI (0.43) | | PHKB | Liver Glycogenosis |
| **12:52676305:T:G** | 4.41E-04 | 4.57 | Offspring-ELLI (0.43) | | KRT1 | Epidermolytic Hyperkeratosis |
| **2:189579904:C:G** | 5.34E-04 | 0.6517 | Offspring-ELLI (0.43) | | SLC40A1 | HFE-Related Haemochromatosis Modifier |
| **2:169637353:A:G** | 8.95E-04 | 0.6991 | Offspring-ELLI (0.43) | | PPIG | Leukaemia |
| **16:3769293:C:T** | 1.35E-03 | 4.556 | Offspring-ELLI (0.43) | | CREBBP | Rubinstein-Taybi Syndrome |
| **2:178725602:C:T** | 1.48E-03 | 0 | Offspring-ELLI (0.43) | | TTN | Cardiomyopathy |
| **19:44908684:T:C** | 1.96E-03 | 0.5905 | Offspring-ELLI (0.43) | | APOE | Apolipoprotein E Deficiency |
| **1:209790735:C:T** | 1.97E-03 | 2.726 | Offspring-ELLI (0.43) | | IRF6 | Cleft Lip/Palate |
| **20:14326373:T:G** | 2.73E-03 | 12.08 | Offspring-ELLI (0.43) | | FLRT3 | Schizophrenia |
| **14:104945801:A:G** | 3.03E-03 | 0.7599 | Offspring-ELLI (0.43) | | AHNAK2 | Schizophrenia |
| **2:233729157:T:C** | 1.20E-04 | 0.7176 | Control-Offspring | | UGT1A3 | Altered Enzyme Activity |
| **4:188101170:C:T** | 4.53E-04 | 1.731 | Control-Offspring | | TRIML2 | Alzheimer Disease Late-Onset |
| **4:188091655:C:T** | 8.23E-04 | 1.671 | Control-Offspring | | TRIML2 | Alzheimer Disease Late-Onset |
| **17:8143454:C:G** | 1.09E-03 | 0.7276 | Control-Offspring | | PER1 | Increased Sex Hormone-Binding Globulin Levels |
| **17:78134494:A:T** | 1.70E-03 | 1.313 | Control-Offspring | | TMC8 | Epidermodysplasia Verruciformis Susceptibility in HIV |
| **14:56805348:G:A** | 1.75E-03 | 0.3133 | Control-Offspring | | OTX2 | Bipolar Disorder |
| **10:79946568:A:G** | 2.40E-03 | 0.7662 | Control-Offspring | | SFTPD | Severe RSV Infection |
| **9:104800523:T:C** | 2.53E-03 | 0.7396 | Control-Offspring | | ABCA1 | Altered HDL Cholesterol Levels |
| **3:128486108:C:T** | 2.64E-03 | 1.364 | Control-Offspring | | GATA2 | Coronary Artery Disease |
| **2:120989968:A:G** | 2.76E-03 | 0.2908 | Control-Offspring | | GLI2 | Holoprosencephaly-Like Phenotype |


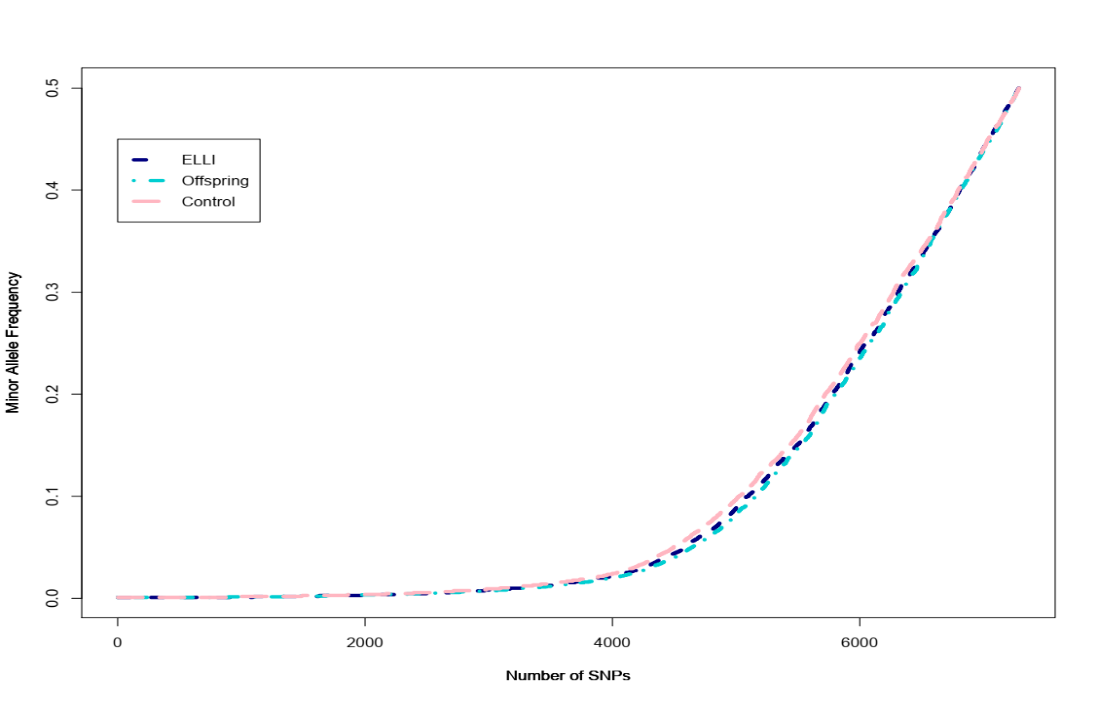


**Supporting Figure** **11: Minor allele frequency** for each group. Mean MAF of ELLI = 0.09, STDV = ±0.14; Mean MAF of offspring = 0.09 STDV = ±0.14; Mean MAF of control = 0.1, STDV = ±0.14.

**Supporting Figure 12:** **Proportion of SNPs at each MAF** “bin” for each group. Minor allele frequency was calculated for the less common allele in each tested population. Differences between groups tested using χ^2^ and found non-significant.


**Supporting Table 4:** Statistical analysis for pathogenic variants in each age-associated disease.

| Disease | Genotype | Group | Mean | SEM | Median | q1 | q3 | IQR (Q3 – Q1) | Kruskal-­‐Wallis test, P-value | Dunn Test, Bonferroni adjusted 2-tailed p-value |
| --- | --- | --- | --- | --- | --- | --- | --- | --- | --- | --- |
| T2D | **Heterozygous** | Control | 2.355263 | 0.058714 | 2 | 1 | 3 | 2 | 0.6007 | - |
|  |  | Offspring | 2.397112 | 0.04668 | 2 | 1 | 3 | 2 |  | - |
|  |  | ELLI | 2.330097 | 0.059078 | 2 | 1 | 3 | 2 |  | - |
|  | **Homozygous** | Control | 0.419173 | 0.026576 | 0 | 0 | 1 | 1 | 0.181 | - |
|  |  | Offspring | 0.438026 | 0.021082 | 0 | 0 | 1 | 1 |  | - |
|  |  | ELLI | 0.491262 | 0.028744 | 0 | 0 | 1 | 1 |  | - |
| Stroke | **Heterozygous** | Control | 3.328947 | 0.061528 | 3 | 2 | 4 | 2 | 0.8407 | - |
|  |  | Offspring | 3.359807 | 0.049462 | 3 | 2 | 4 | 2 |  | - |
|  |  | ELLI | 3.376699 | 0.064607 | 3 | 2 | 4 | 2 |  | - |
|  | **Homozygous** | Control | 0.806391 | 0.03879 | 1 | 0 | 1 | 1 | 0.5691 | - |
|  |  | Offspring | 0.824308 | 0.028655 | 1 | 0 | 1 | 1 |  | - |
|  |  | ELLI | 0.790291 | 0.035699 | 1 | 0 | 1 | 1 |  | - |
| Parkinson's disease | **Heterozygous** | Control | 8.317669 | 0.098509 | 8 | 7 | 10 | 3 | 0.3917 | - |
|  |  | Offspring | 8.359807 | 0.080696 | 8 | 7 | 10 | 3 |  | - |
|  |  | ELLI | 8.201942 | 0.099657 | 8 | 7 | 10 | 3 |  | - |
|  | **Homozygous** | Control | 1.731203 | 0.0535 | 2 | 1 | 3 | 2 | 0.9816 | - |
|  |  | Offspring | 1.753309 | 0.044084 | 2 | 1 | 2 | 1 |  | - |
|  |  | ELLI | 1.728155 | 0.053651 | 2 | 1 | 2 | 1 |  | - |
| Myocardial infarction | **Heterozygous** | Control | 9.832707 | 0.116622 | 10 | 8 | 12 | 4 | 0.1894 | - |
|  |  | Offspring | 10.135981 | 0.096265 | 10 | 8 | 12 | 4 |  | - |
|  |  | ELLI | 9.831068 | 0.113104 | 10 | 8 | 12 | 4 |  | - |
|  | **Homozygous** | Control | 2.298872 | 0.065703 | 2 | 1 | 3 | 2 | 0.221 | - |
|  |  | Offspring | 2.43562 | 0.054148 | 2 | 1 | 3 | 2 |  | - |
|  |  | ELLI | 2.436893 | 0.069066 | 2 | 1 | 3 | 2 |  | - |
| Dementia | **Heterozygous** | Control | 1.889098 | 0.048644 | 2 | 1 | 3 | 2 | 0.8401 | - |
|  |  | Offspring | 1.906137 | 0.037243 | 2 | 1 | 3 | 2 |  | - |
|  |  | ELLI | 1.873786 | 0.044948 | 2 | 1 | 3 | 2 |  | - |
|  | **Homozygous** | Control | 0.445489 | 0.027227 | 0 | 0 | 1 | 1 | 0.773 | - |
|  |  | Offspring | 0.451264 | 0.0218 | 0 | 0 | 1 | 1 |  | - |
|  |  | ELLI | 0.475728 | 0.028727 | 0 | 0 | 1 | 1 |  | - |
| Non-myocardial infarction CVDs | **Heterozygous** | Control | 1.469925 | 0.037935 | 1 | 1 | 2 | 1 | 0.9069 | - |
|  |  | Offspring | 1.45006 | 0.03116 | 1 | 1 | 2 | 1 |  | - |
|  |  | ELLI | 1.475728 | 0.040605 | 1 | 1 | 2 | 1 |  | - |
|  | **Homozygous** | Control | 0.473684 | 0.027947 | 0 | 0 | 1 | 1 | 0.8191 | - |
|  |  | Offspring | 0.476534 | 0.021717 | 0 | 0 | 1 | 1 |  | - |
|  |  | ELLI | 0.442718 | 0.025569 | 0 | 0 | 1 | 1 |  | - |
| COPD | **Heterozygous** | Control | 3.68797 | 0.070754 | 4 | 2 | 5 | 3 | 0.2469 | - |
|  |  | Offspring | 3.566787 | 0.057116 | 4 | 2 | 5 | 3 |  | - |
|  |  | ELLI | 3.708738 | 0.073764 | 4 | 3 | 5 | 2 |  | - |
|  | **Homozygous** | Control | 0.819549 | 0.036516 | 1 | 0 | 1 | 1 | 0.3726 | - |
|  |  | Offspring | 0.872443 | 0.029896 | 1 | 0 | 1 | 1 |  | - |
|  |  | ELLI | 0.813592 | 0.037392 | 1 | 0 | 1 | 1 |  | - |
| Cancer | **Heterozygous** | Control | 106.4774 | 0.402154 | 106.5 | 100.8 | 113 | 12.2 | 0.02744 | ELLI - Ctrl: 0.0140* |
|  |  | Offspring | 105.5355 | 0.315493 | 105 | 99 | 112 | 13 |  | ELLI - Off: 0.5463 |
|  |  | ELLI | 105.0505 | 0.405269 | 105 | 99 | 111 | 12 |  | Ctrl - Off: 0.0721 |
|  | **Homozygous** | Control | 24.14286 | 0.223152 | 24 | 20.75 | 27 | 6.25 | 0.09464 | - |
|  |  | Offspring | 24.30566 | 0.180712 | 24 | 21 | 28 | 7 |  | - |
|  |  | ELLI | 24.82718 | 0.232178 | 24 | 21 | 28 | 7 |  | - |
| Alzheimer's disease | **Heterozygous** | Control | 22.23872 | 0.186509 | 22 | 19 | 25 | 6 | 0.01904 | ELLI - Ctrl: 0.0840 |
|  |  | Offspring | 22.43803 | 0.138605 | 22 | 20 | 25 | 5 |  | ELLI - Off: 0.0081* |
|  |  | ELLI | 21.87573 | 0.174928 | 22 | 19 | 24 | 5 |  | Ctrl - Off: 0.7421 |
|  | **Homozygous** | Control | 4.734962 | 0.091006 | 5 | 3 | 6 | 3 | 0.1807 | - |
|  |  | Offspring | 4.925391 | 0.07252 | 5 | 4 | 6 | 2 |  | - |
|  |  | ELLI | 4.862136 | 0.092342 | 5 | 3 | 6 | 3 |  | - |

**Supporting Table** **6:** Statistical analysis for pathogenic variants in different modes of inheritance.

| Type of inheritance | Group | Median number of Heterozygous | Kruskal-Walis | Median number of Homozygous | Kruskal-Walis |
| --- | --- | --- | --- | --- | --- |
| AR | ELLI | 1 | p=0.66 | 0 | p=0.08 |
|  | Offspring | 1 |  | 0 |  |
|  | Control | 1 |  | 0 |  |
| AD | ELLI | 0 | P=0.77 | 0 | P=0.06 |
|  | Offspring | 0 |  | 0 |  |
|  | Control | 0 |  | 0 |  |
| AR/AD | ELLI | 1 | P=0.36 | 0 | P=0.24 |
|  | Offspring | 1 |  | 0 |  |
|  | Control | 1 |  | 0 |  |


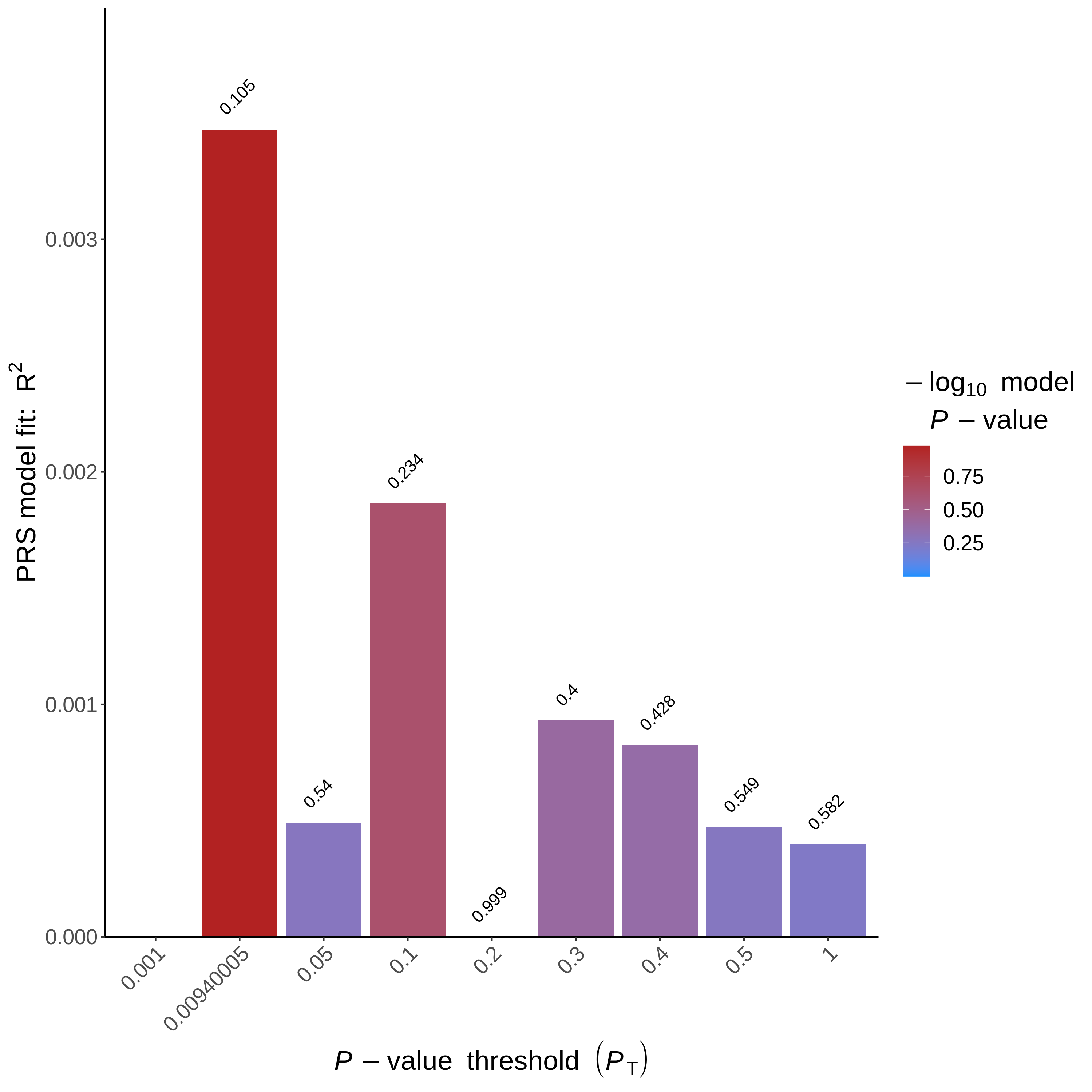

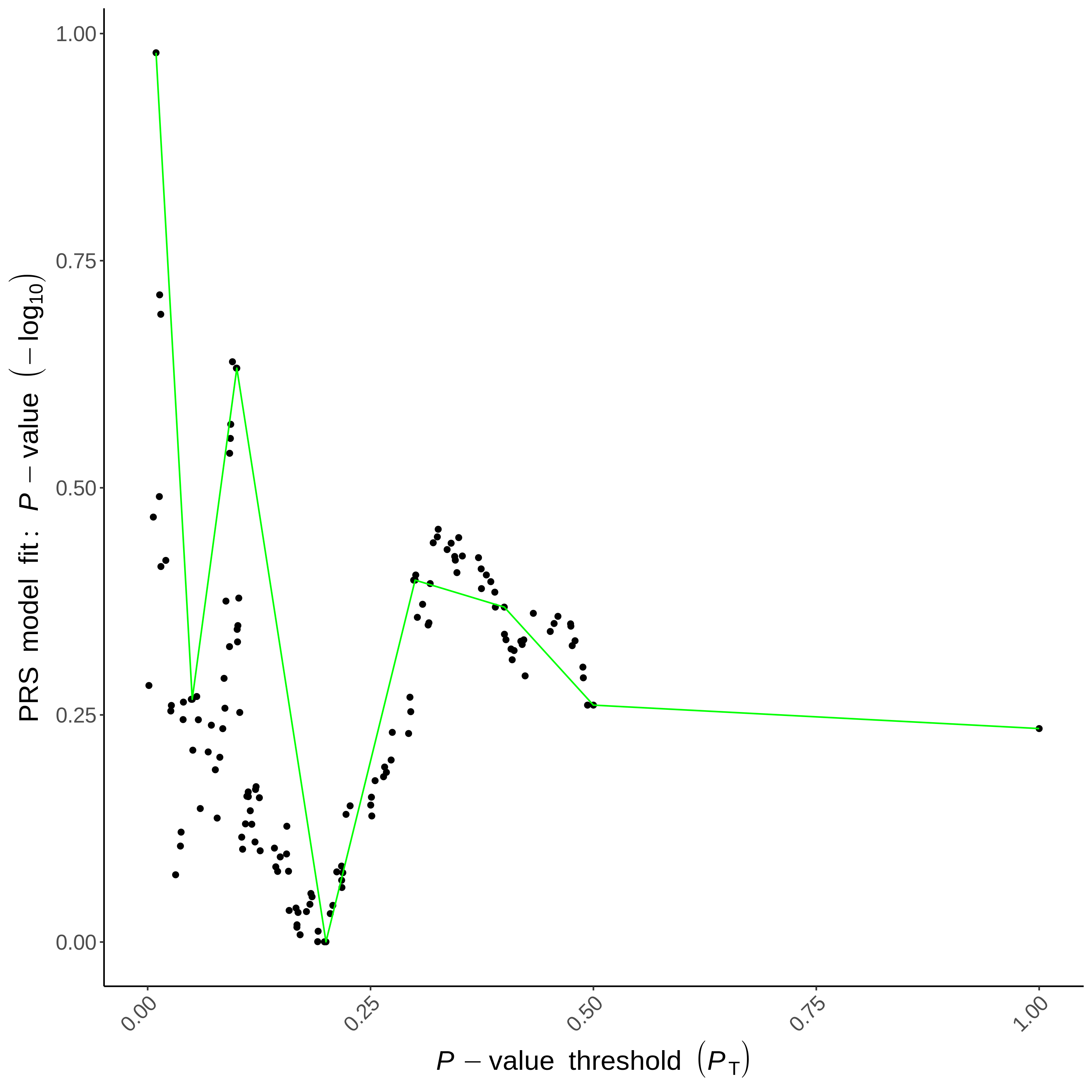


**A**

**B**

**Supporting Figure** **13: Polygenic risk scores of control-ELLI case-control analysis.**  Performed using PRSice using CHARGE cohort as a discovery set and the case-control analysis of control vs. ELLI as the target set. **A.** Barplot showing predictive value (R^2^) in the control-ELLI sample based on SHNPs with p-values below the threshold in the CHARGE discovery cohort. **B.** Scatter plot showing the p-values of the predictive effect in black and an aggregated trend line in green.


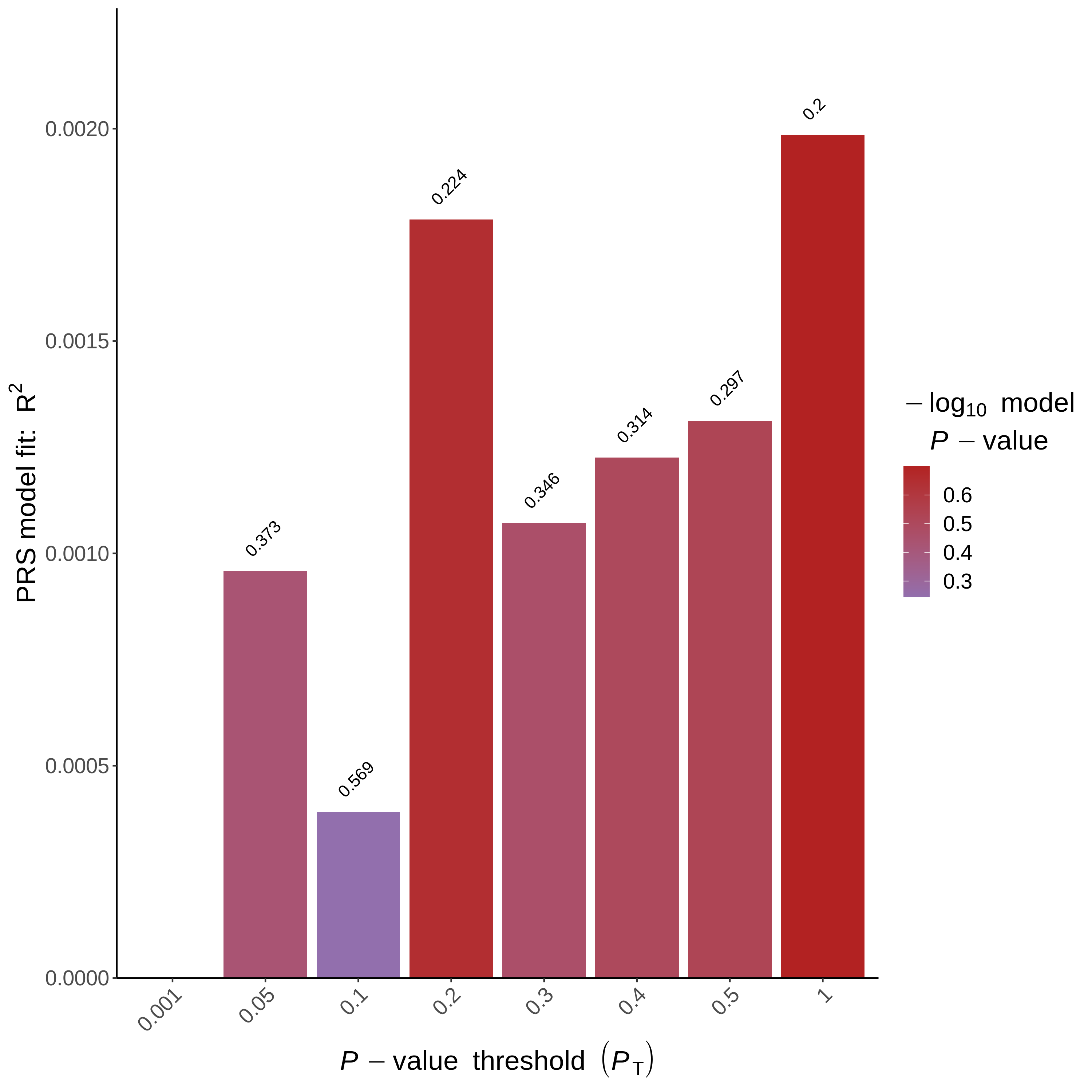

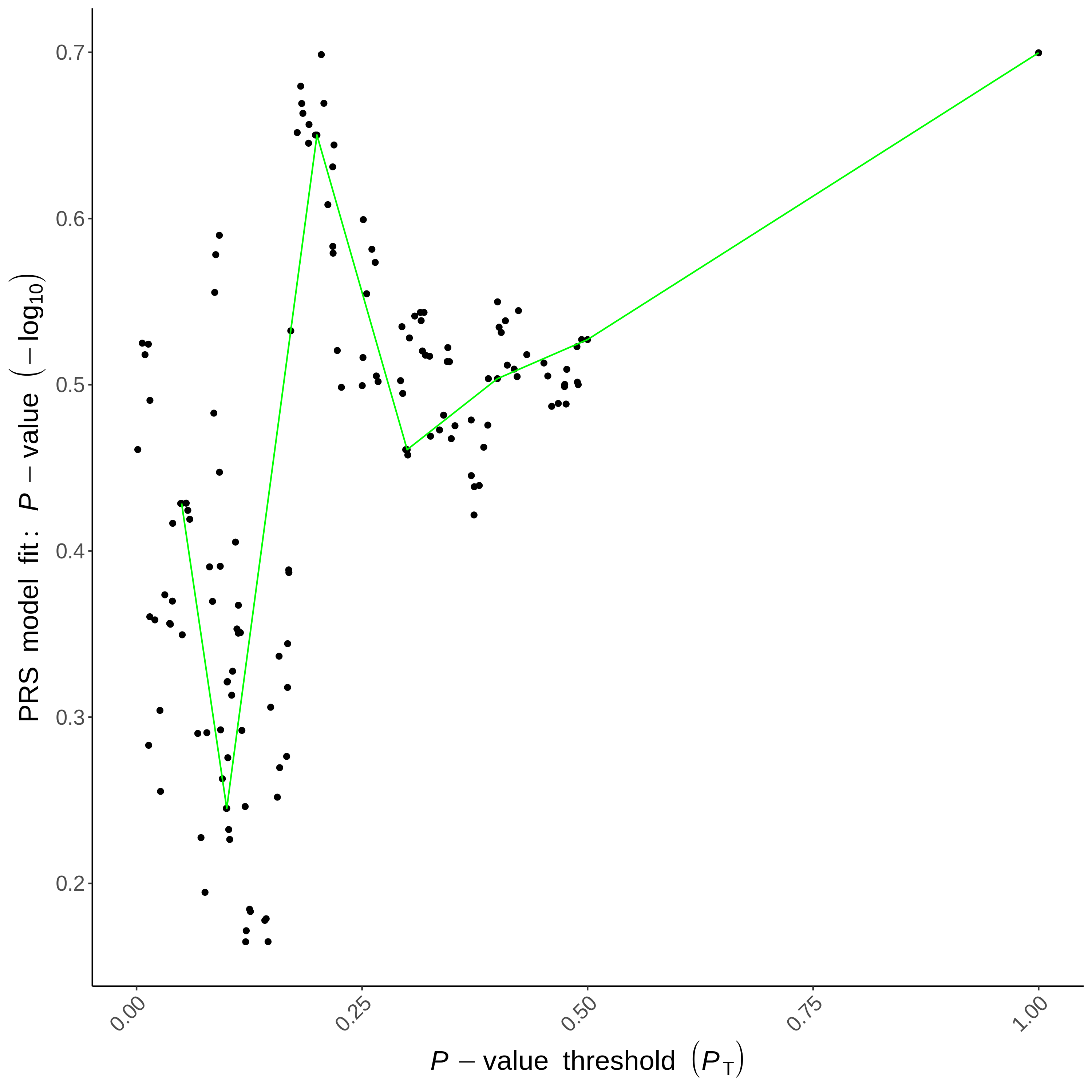


**A**

**B**

**Supporting Figure** **14: Polygenic risk scores of control-offspring case-control analysis.**  Performed using PRSice using CHARGE cohort as a discovery set and the case-control analysis of control vs. offspring as the target set. **A.** Barplot showing predictive value (R^2^) in the control-offspring sample based on SHNPs with p-values below the threshold in the CHARGE discovery cohort. **B.** Scatter plot showing the p-values of the predictive effect in black and an aggregated trend line in green.


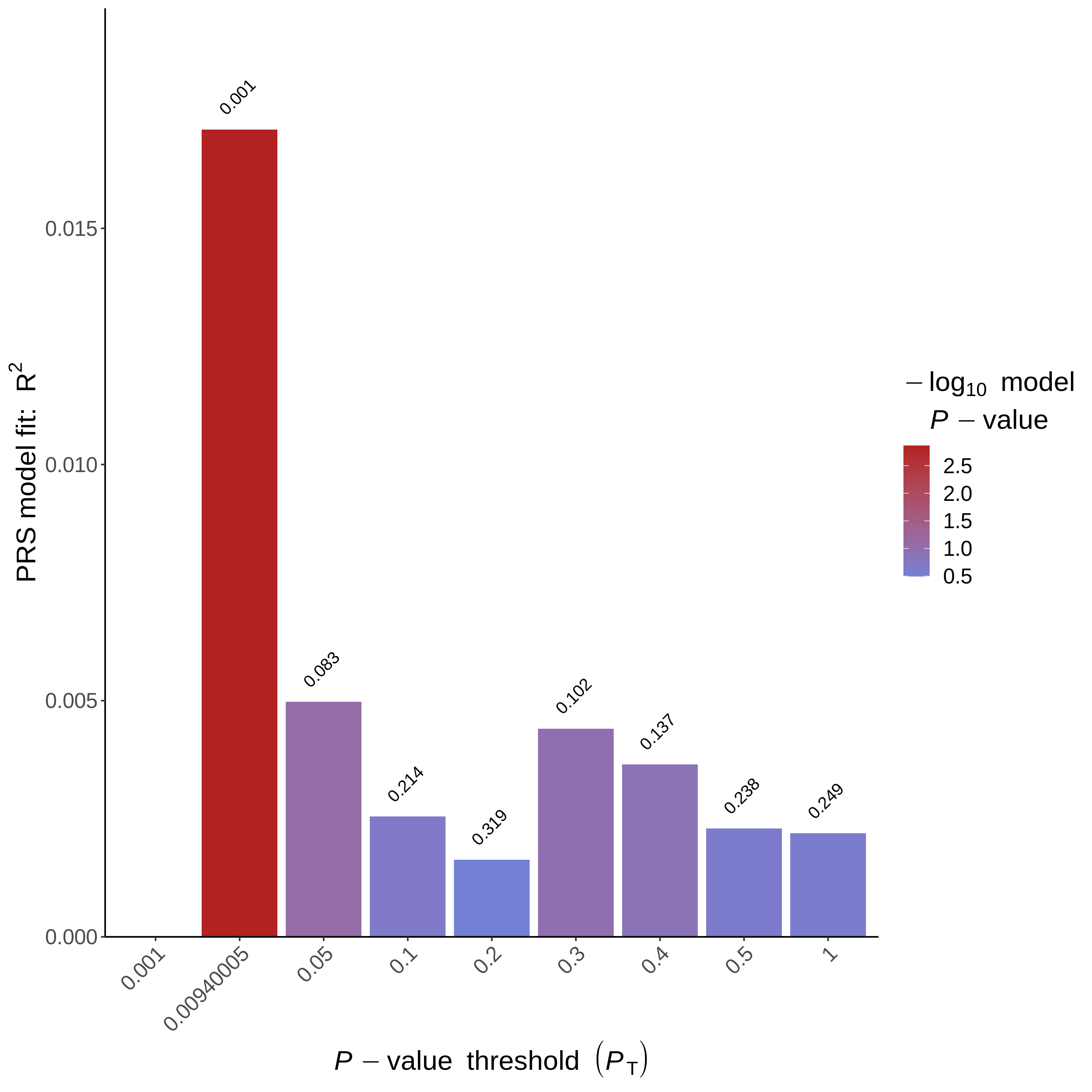

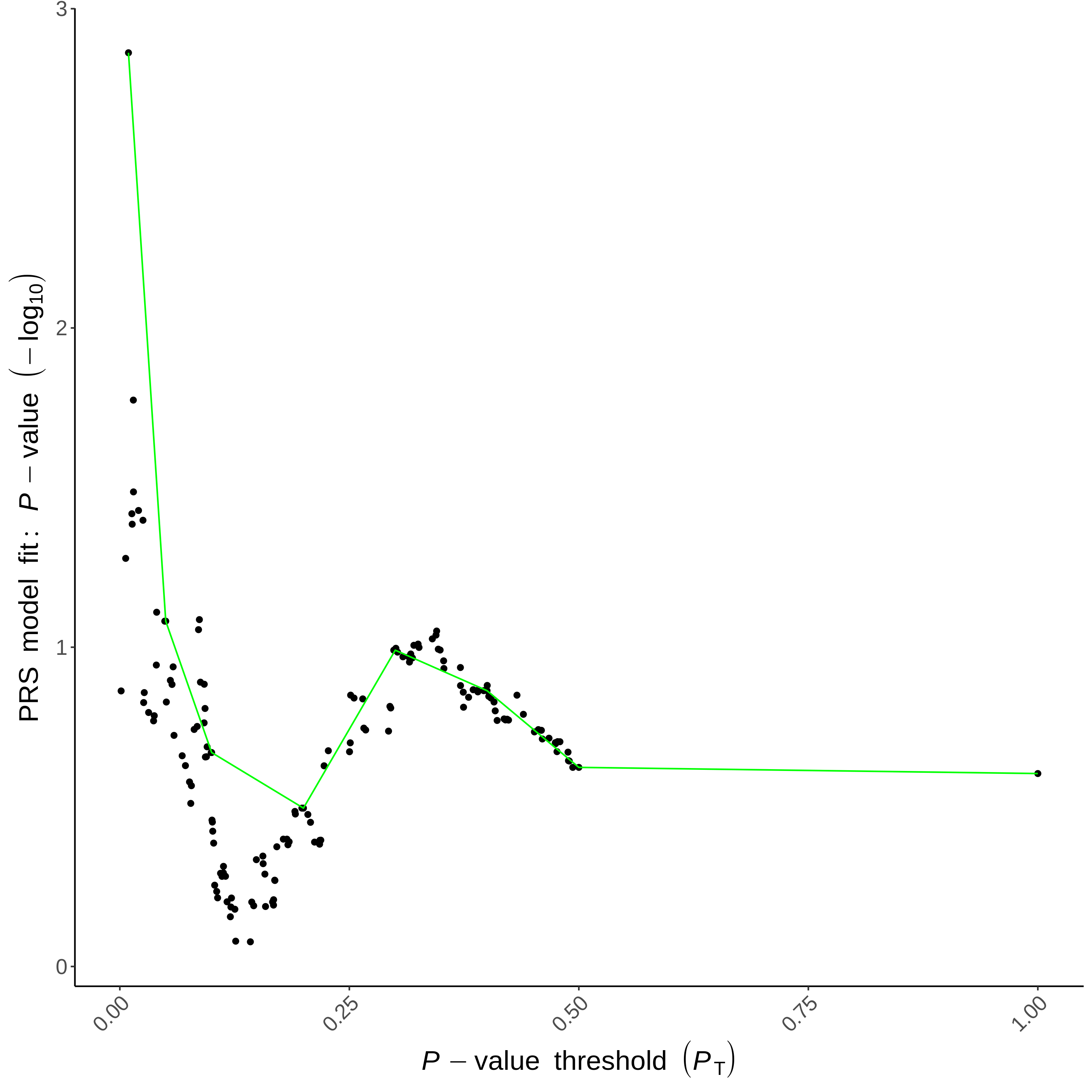


**A**

**B**

**Supporting Figure** **15: Polygenic risk scores of offspring-ELLI case-control analysis.**  Performed using PRSice using CHARGE cohort as a discovery set and the case-control analysis of offspring vs. ELLI as the target set. **A.** Barplot showing predictive value (R^2^) in the offspring-ELLI sample based on SHNPs with p-values below the threshold in the CHARGE discovery cohort. **B.** Scatter plot showing the p-values of the predictive effect in black and an aggregated trend line in green.

**Supporting Table 7:** List of SNPs used for PRS analysis.

| **Analysis** | **CHR** | **Position** | **Effective Allele** | **Alternative Allele** | **Weight** |
| --- | --- | --- | --- | --- | --- |
| **Common to all 3** | 11 | 126473292 | T | C | 0.00138 |
|  | 19 | 965043 | T | C | 0.006328 |
|  | 19 | 49857801 | A | G | 0.009387 |
|  | 9 | 6551009 | A | C | 0.01307 |
|  | 11 | 219089 | T | C | 0.01343 |
|  | 9 | 712156 | T | G | 0.0147 |
|  | 9 | 2077875 | T | C | 0.01483 |
|  | 19 | 33114406 | T | C | 0.02033 |
|  | 19 | 633381 | A | G | 0.02593 |
|  | 9 | 732302 | T | C | 0.02656 |
|  | 18 | 694206 | A | G | 0.03135 |
|  | 9 | 6757688 | A | G | 0.03673 |
|  | 9 | 8518143 | T | C | 0.03746 |
|  | 9 | 154795 | T | C | 0.03971 |
|  | 9 | 5713832 | T | G | 0.04009 |
|  | 21 | 40708853 | T | C | 0.04895 |
|  | 6 | 656343 | T | C | 0.05062 |
|  | 11 | 419706 | T | C | 0.05504 |
|  | 9 | 7046917 | A | G | 0.05684 |
|  | 9 | 2039983 | A | C | 0.05897 |
|  | 11 | 828784 | A | G | 0.06786 |
|  | 9 | 4576680 | T | C | 0.07145 |
|  | 21 | 43795165 | T | C | 0.07588 |
|  | 9 | 710818 | A | G | 0.07792 |
|  | 11 | 293188 | T | C | 0.08092 |
|  | 9 | 1056101 | T | G | 0.08423 |
|  | 9 | 5969249 | A | G | 0.0857 |
|  | 18 | 672836 | A | G | 0.08661 |
|  | 19 | 55501230 | A | G | 0.08778 |
|  | 9 | 325817 | T | C | 0.09178 |
|  | 11 | 614367 | T | C | 0.09191 |
|  | 18 | 192802 | A | G | 0.09277 |
|  | 9 | 5770047 | A | G | 0.09317 |
|  | 19 | 731144 | A | G | 0.09507 |
|  | 11 | 280221 | A | C | 0.09948 |
|  | 2 | 169242963 | T | C | 0.1004 |
|  | 3 | 129575922 | T | C | 0.1008 |
|  | 9 | 2191246 | T | C | 0.1012 |
|  | 15 | 100063782 | A | G | 0.1022 |
|  | 6 | 292658 | T | C | 0.1033 |
|  | 19 | 990394 | T | C | 0.1055 |
|  | 9 | 8497250 | T | C | 0.1065 |
|  | 11 | 249097 | A | G | 0.1097 |
| **Common to all 3** | 19 | 804079 | T | C | 0.1113 |
|  | 11 | 123943771 | A | G | 0.1128 |
|  | 5 | 128361896 | A | G | 0.1129 |
|  | 9 | 8465598 | A | G | 0.1151 |
|  | 9 | 273160 | T | C | 0.1168 |
|  | 2 | 277003 | A | G | 0.1204 |
|  | 19 | 735017 | T | C | 0.121 |
|  | 8 | 81283316 | A | C | 0.1216 |
|  | 11 | 308178 | T | C | 0.1253 |
|  | 7 | 132179620 | A | G | 0.1262 |
|  | 5 | 179642412 | A | G | 0.1422 |
|  | 9 | 707064 | A | G | 0.1437 |
|  | 7 | 73840130 | T | G | 0.1458 |
|  | 11 | 1411461 | T | C | 0.1487 |
|  | 9 | 7013909 | A | G | 0.1561 |
|  | 6 | 488905 | T | C | 0.158 |
|  | 18 | 480801 | A | C | 0.1587 |
|  | 6 | 30170699 | T | C | 0.1664 |
|  | 9 | 417990 | T | C | 0.1674 |
|  | 9 | 327996 | T | C | 0.1675 |
|  | 9 | 6255881 | T | G | 0.1687 |
|  | 18 | 3874343 | T | C | 0.171 |
|  | 11 | 419167 | T | G | 0.1781 |
|  | 12 | 21022757 | A | G | 0.1781 |
|  | 12 | 123309475 | T | C | 0.182 |
|  | 9 | 6814821 | A | G | 0.1831 |
|  | 8 | 144504245 | A | G | 0.1844 |
|  | 9 | 2622121 | A | G | 0.1907 |
|  | 9 | 4688433 | T | C | 0.1912 |
|  | 11 | 75728805 | A | C | 0.1983 |
|  | 19 | 549829 | A | C | 0.2048 |
|  | 1 | 153543963 | A | G | 0.2077 |
|  | 9 | 317218 | A | C | 0.2121 |
|  | 19 | 885818 | A | G | 0.2175 |
|  | 9 | 4844704 | T | C | 0.2176 |
|  | 22 | 42623718 | A | C | 0.2179 |
|  | 11 | 557540 | A | G | 0.2189 |
|  | 16 | 56343836 | A | G | 0.2225 |
|  | 19 | 407900 | A | G | 0.2271 |
|  | 7 | 92327765 | T | C | 0.2502 |
|  | 5 | 72186768 | T | C | 0.251 |
|  | 9 | 6015011 | T | C | 0.2514 |
|  | 19 | 652948 | T | G | 0.2551 |
|  | 11 | 74302317 | T | C | 0.2646 |
|  | 19 | 929753 | A | G | 0.2658 |
| **Common to all 3** | 8 | 143795592 | A | G | 0.2678 |
|  | 22 | 37647572 | A | G | 0.2927 |
|  | 18 | 158795 | A | G | 0.2942 |
|  | 19 | 843692 | A | G | 0.2951 |
|  | 8 | 132757379 | T | C | 0.2984 |
|  | 2 | 189053032 | T | C | 0.3007 |
|  | 11 | 400040 | A | G | 0.3025 |
|  | 11 | 1021268 | T | C | 0.3084 |
|  | 18 | 214959 | A | G | 0.3146 |
|  | 9 | 5381870 | A | G | 0.3155 |
|  | 9 | 429940 | T | G | 0.3169 |
|  | 11 | 1085368 | A | G | 0.3203 |
|  | 1 | 158844029 | A | G | 0.3249 |
|  | 9 | 8338878 | A | C | 0.3259 |
|  | 2 | 238332917 | T | C | 0.3404 |
|  | 17 | 75572929 | T | C | 0.3444 |
|  | 9 | 214908 | T | C | 0.3451 |
|  | 2 | 214767532 | A | G | 0.3469 |
|  | 11 | 124424807 | T | C | 0.3489 |
|  | 14 | 77026917 | A | G | 0.353 |
|  | 9 | 5787647 | A | G | 0.3711 |
|  | 1 | 32771372 | T | C | 0.3741 |
|  | 11 | 709401 | A | G | 0.3744 |
|  | 11 | 830120 | A | G | 0.3799 |
|  | 17 | 39922537 | A | G | 0.3849 |
|  | 9 | 2081739 | A | G | 0.3894 |
|  | 9 | 8485686 | T | C | 0.39 |
|  | 6 | 576902 | T | C | 0.4002 |
|  | 3 | 64568287 | T | C | 0.4019 |
|  | 11 | 1025987 | A | G | 0.4089 |
|  | 18 | 335099 | T | C | 0.411 |
|  | 9 | 4625429 | T | C | 0.4186 |
|  | 10 | 103368208 | T | C | 0.4219 |
|  | 19 | 719615 | T | C | 0.4234 |
|  | 11 | 199673 | T | C | 0.4326 |
|  | 9 | 376929 | A | G | 0.4516 |
|  | 12 | 14935047 | A | G | 0.4559 |
|  | 9 | 2651340 | A | G | 0.4602 |
|  | 19 | 344053 | T | C | 0.4744 |
|  | 20 | 54173104 | A | G | 0.4747 |
|  | 9 | 741017 | A | G | 0.4762 |
|  | 9 | 3394739 | A | C | 0.4882 |
|  | 16 | 77817102 | A | G | 0.4887 |
|  | 19 | 498524 | A | G | 0.4934 |
|  | 1 | 30714190 | T | C | 0.874 |
| **Common to all 3** | 1 | 161631002 | A | C | 0.609 |
|  | 1 | 173834605 | A | G | 0.6934 |
|  | 1 | 212732289 | A | G | 0.8191 |
|  | 2 | 672745 | T | C | 0.9557 |
|  | 2 | 11458429 | T | C | 0.8759 |
|  | 2 | 32442494 | A | G | 0.5394 |
|  | 2 | 178704709 | A | G | 0.9128 |
|  | 3 | 12820087 | T | C | 0.5629 |
|  | 3 | 33852440 | T | C | 0.956 |
|  | 3 | 195752385 | T | C | 0.786 |
|  | 3 | 195885589 | T | C | 0.8908 |
|  | 4 | 1108407 | A | G | 0.7749 |
|  | 4 | 10444650 | T | C | 0.795 |
|  | 4 | 169426096 | A | G | 0.9904 |
|  | 5 | 1225449 | T | C | 0.6796 |
|  | 6 | 397290 | T | C | 0.5691 |
|  | 6 | 564145 | A | G | 0.5075 |
|  | 6 | 599209 | T | C | 0.8226 |
|  | 6 | 3010103 | A | G | 0.9737 |
|  | 6 | 31355366 | T | C | 0.9095 |
|  | 6 | 32302541 | T | G | 0.6069 |
|  | 6 | 34028094 | A | G | 0.5447 |
|  | 6 | 34540913 | A | G | 0.7778 |
|  | 6 | 47025899 | A | G | 0.6192 |
|  | 6 | 132772949 | T | C | 0.9572 |
|  | 6 | 168310490 | A | G | 0.8165 |
|  | 7 | 195501 | A | G | 0.715 |
|  | 7 | 105678109 | T | C | 0.9368 |
|  | 7 | 155644781 | T | C | 0.5879 |
|  | 8 | 11332026 | T | C | 0.9692 |
|  | 8 | 142912905 | T | C | 0.5452 |
|  | 9 | 162541 | A | G | 0.7621 |
|  | 9 | 214804 | A | G | 0.8541 |
|  | 9 | 215086 | A | C | 0.9895 |
|  | 9 | 215296 | A | G | 0.6126 |
|  | 9 | 379965 | T | C | 0.9196 |
|  | 9 | 404864 | A | G | 0.9367 |
|  | 9 | 432081 | T | C | 0.7368 |
|  | 9 | 732629 | T | C | 0.6867 |
|  | 9 | 847208 | A | G | 0.5538 |
|  | 9 | 894036 | T | C | 0.7438 |
|  | 9 | 989943 | T | C | 0.7214 |
|  | 9 | 2651809 | T | C | 0.7128 |
|  | 9 | 2717676 | T | G | 0.5232 |
|  | 9 | 2804393 | A | G | 0.7894 |
| **Common to all 3** | 9 | 2824902 | T | C | 0.8296 |
|  | 9 | 3247500 | A | G | 0.524 |
|  | 9 | 3400277 | A | G | 0.64 |
|  | 9 | 4544640 | T | C | 0.9481 |
|  | 9 | 4572480 | T | C | 0.5281 |
|  | 9 | 4625415 | T | C | 0.7775 |
|  | 9 | 4662394 | A | G | 0.6397 |
|  | 9 | 4679906 | A | G | 0.7148 |
|  | 9 | 4834299 | T | G | 0.5859 |
|  | 9 | 5122932 | A | G | 0.948 |
|  | 9 | 5557708 | T | C | 0.6316 |
|  | 9 | 5772537 | T | C | 0.9741 |
|  | 9 | 6329888 | A | G | 0.7789 |
|  | 9 | 6594978 | T | C | 0.6435 |
|  | 9 | 6606545 | T | C | 0.7018 |
|  | 9 | 6924918 | T | G | 0.758 |
|  | 9 | 6984479 | A | G | 0.6543 |
|  | 9 | 7015793 | T | C | 0.672 |
|  | 9 | 7076371 | T | C | 0.7749 |
|  | 9 | 7170742 | A | G | 0.9113 |
|  | 9 | 12698363 | T | C | 0.8688 |
|  | 9 | 12698471 | T | C | 0.8248 |
|  | 9 | 15472411 | A | G | 0.829 |
|  | 9 | 35906601 | A | C | 0.8713 |
|  | 9 | 104837095 | A | G | 0.9434 |
|  | 9 | 110431847 | A | G | 0.9346 |
|  | 10 | 26751583 | A | G | 0.7032 |
|  | 10 | 97679939 | A | G | 0.8391 |
|  | 11 | 199813 | A | G | 0.9199 |
|  | 11 | 205889 | T | C | 0.699 |
|  | 11 | 299586 | A | G | 0.9554 |
|  | 11 | 392634 | T | C | 0.9545 |
|  | 11 | 397732 | T | G | 0.7205 |
|  | 11 | 495103 | A | G | 0.6443 |
|  | 11 | 556457 | T | C | 0.7718 |
|  | 11 | 562820 | T | C | 0.5097 |
|  | 11 | 640099 | A | G | 0.5204 |
|  | 11 | 680866 | A | G | 0.7372 |
|  | 11 | 802125 | T | C | 0.9191 |
|  | 11 | 5352508 | A | G | 0.5751 |
|  | 11 | 5708189 | T | C | 0.9137 |
|  | 11 | 11332799 | T | C | 0.9973 |
|  | 12 | 133007053 | A | G | 0.665 |
|  | 13 | 108270266 | A | G | 0.6586 |
|  | 14 | 36680380 | T | C | 0.6583 |
| **Common to all 3** | 15 | 72168908 | T | C | 0.5617 |
|  | 15 | 100905610 | A | G | 0.7321 |
|  | 16 | 19546503 | A | G | 0.5781 |
|  | 16 | 89193390 | T | C | 0.7632 |
|  | 16 | 89698070 | T | C | 0.8505 |
|  | 17 | 1079174 | A | G | 0.6926 |
|  | 17 | 50139629 | T | C | 0.8103 |
|  | 18 | 204780 | A | G | 0.942 |
|  | 18 | 247834 | T | G | 0.6364 |
|  | 18 | 334994 | A | G | 0.6475 |
|  | 18 | 357570 | T | C | 0.9128 |
|  | 18 | 580623 | T | C | 0.5849 |
|  | 18 | 618124 | T | C | 0.747 |
|  | 18 | 674320 | A | G | 0.6649 |
|  | 18 | 675307 | A | G | 0.7687 |
|  | 18 | 712263 | T | G | 0.6071 |
|  | 18 | 745884 | A | C | 0.8698 |
|  | 18 | 3656058 | T | C | 0.7844 |
|  | 19 | 288246 | T | C | 0.6189 |
|  | 19 | 312313 | A | G | 0.8111 |
|  | 19 | 538930 | A | G | 0.9794 |
|  | 19 | 582468 | A | G | 0.8221 |
|  | 19 | 651543 | A | G | 0.5753 |
|  | 19 | 652238 | A | G | 0.6347 |
|  | 19 | 687279 | T | C | 0.9576 |
|  | 19 | 691909 | T | C | 0.7389 |
|  | 19 | 727550 | T | C | 0.7202 |
|  | 19 | 740560 | T | C | 0.5904 |
|  | 19 | 757412 | A | G | 0.5677 |
|  | 19 | 829723 | A | G | 0.7245 |
|  | 19 | 872089 | A | G | 0.8268 |
|  | 19 | 971933 | A | G | 0.7202 |
|  | 19 | 14799842 | T | C | 0.859 |
|  | 19 | 35164248 | T | C | 0.7077 |
|  | 19 | 54278817 | A | G | 0.6835 |
|  | 19 | 54594897 | T | C | 0.5756 |
| **Common to control-ELLI and control-offspring** | 19 | 621063 | A | G | 0.3359 |
|  | 11 | 372095 | T | C | 0.4602 |
|  | 7 | 1000554 | A | C | 0.673 |
|  | 11 | 554003 | T | G | 0.9054 |
|  | 12 | 6941661 | T | C | 0.9876 |
|  | 19 | 869037 | A | G | 0.7256 |
| **Common to control-offspring and offspring-ELLI** | 1 | 58538927 | T | C | 0.1689 |
|  | 11 | 626974 | T | C | 0.3187 |
|  | 11 | 3660412 | A | G | 0.371 |
| **Common to control-offspring and offspring-ELLI** | 15 | 40853666 | T | C | 0.4043 |
|  | 6 | 89154972 | A | C | 0.4675 |
|  | 11 | 292798 | A | G | 0.4769 |
|  | 9 | 21206509 | T | G | 0.4896 |
|  | 9 | 676954 | T | C | 0.7994 |
|  | 11 | 558165 | T | G | 0.7406 |
|  | 20 | 50130865 | A | C | 0.9399 |
| **Common to control-ELLI and offspring-ELLI** | 2 | 241190013 | T | C | 0.1558 |
|  | 9 | 77282239 | A | G | 0.4076 |
|  | 11 | 107336784 | T | C | 0.4201 |
|  | 15 | 74191317 | T | C | 0.4794 |
|  | 9 | 8454566 | T | C | 0.7865 |
|  | 19 | 991892 | T | C | 0.9532 |
|  | 21 | 46126476 | T | C | 0.6768 |
| **Control-ELLI** | 11 | 290891 | T | C | 0.2731 |
|  | 11 | 290816 | A | G | 0.2744 |
|  | 9 | 2729733 | T | C | 0.8292 |
|  | 11 | 721737 | A | G | 0.7484 |
|  | 11 | 802902 | T | G | 0.8395 |
|  | 11 | 1019194 | A | G | 0.8899 |
|  | 19 | 464310 | A | G | 0.6661 |
| **Control-offspring** | 9 | 6330380 | A | C | 0.2609 |
|  | 9 | 3277260 | A | G | 0.5389 |
|  | 10 | 68231322 | T | C | 0.7067 |
| **Offspring-ELLI** | 11 | 1629943 | T | G | 0.0252 |
|  | 12 | 133105565 | T | C | 0.05801 |
|  | 9 | 2123639 | T | C | 0.07723 |
|  | 3 | 42210922 | A | G | 0.09426 |
|  | 19 | 1461863 | A | C | 0.3527 |
|  | 9 | 21367352 | A | G | 0.3964 |
|  | 11 | 1009890 | T | C | 0.4396 |
|  | 11 | 124038487 | T | C | 0.4592 |
|  | 4 | 42049435 | A | G | 0.517 |
|  | 11 | 621826 | T | G | 0.6765 |
|  | 15 | 72709825 | T | C | 0.5379 |
|  | 19 | 620504 | T | C | 0.516 |
