# Supplementary figures and images for: Similar burden of pathogenic coding variants in exceptionally long‐lived individuals and individuals without exceptional longevity
Source: Aging Cell. 2020 Aug 29;19(10):e13216. doi: 10.1111/acel.13216 (PMC7576295; doi:10.1111/acel.13216)

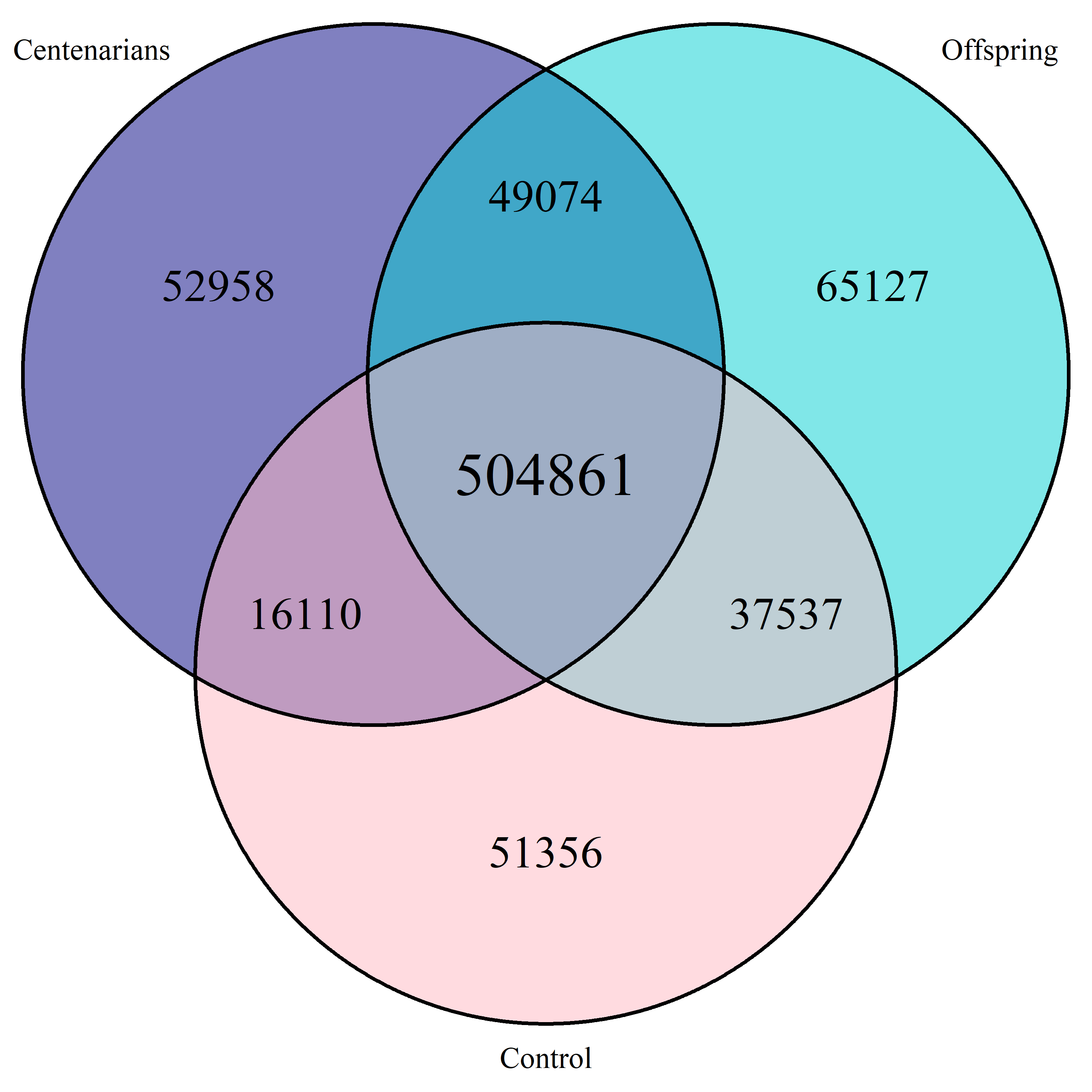

Supplement: Supplementary file 5 [file ACEL-19-e13216-s005.tiff]

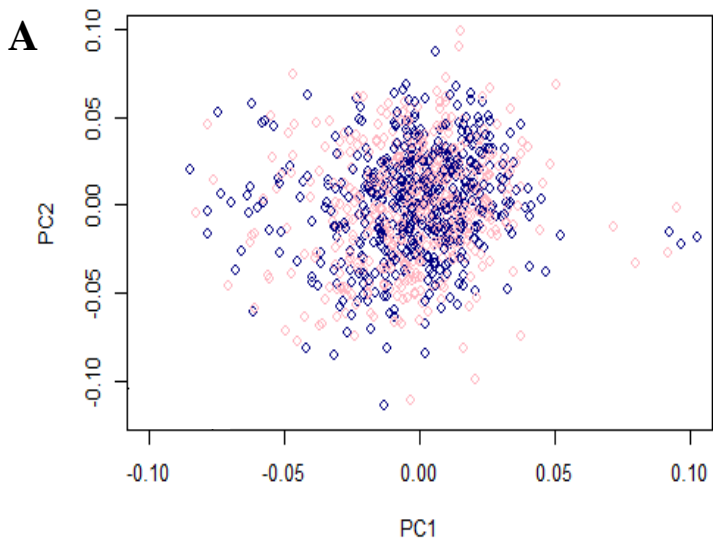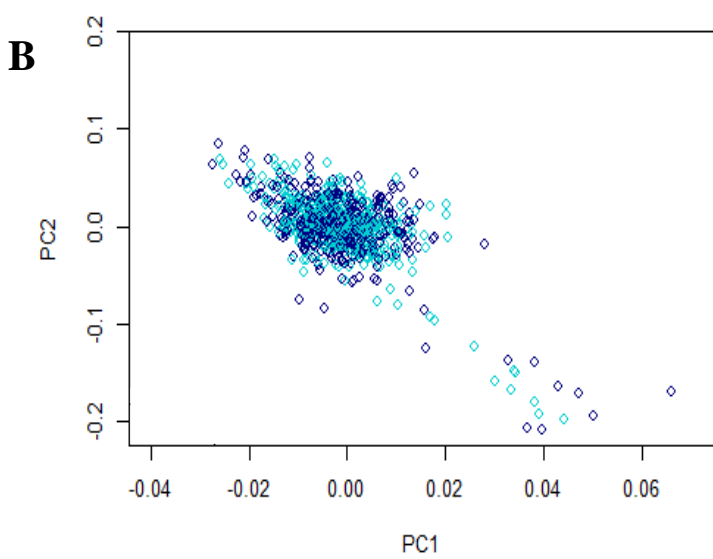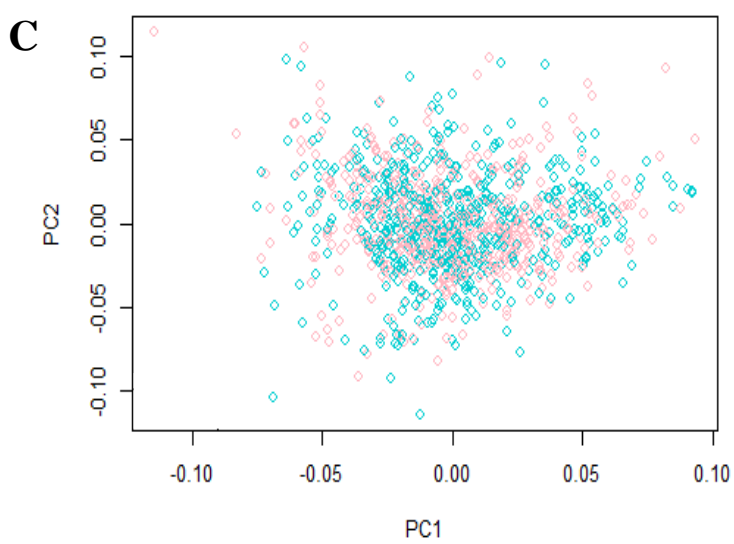

Supplement: Supplementary file 7 [file ACEL-19-e13216-s007.pdf]

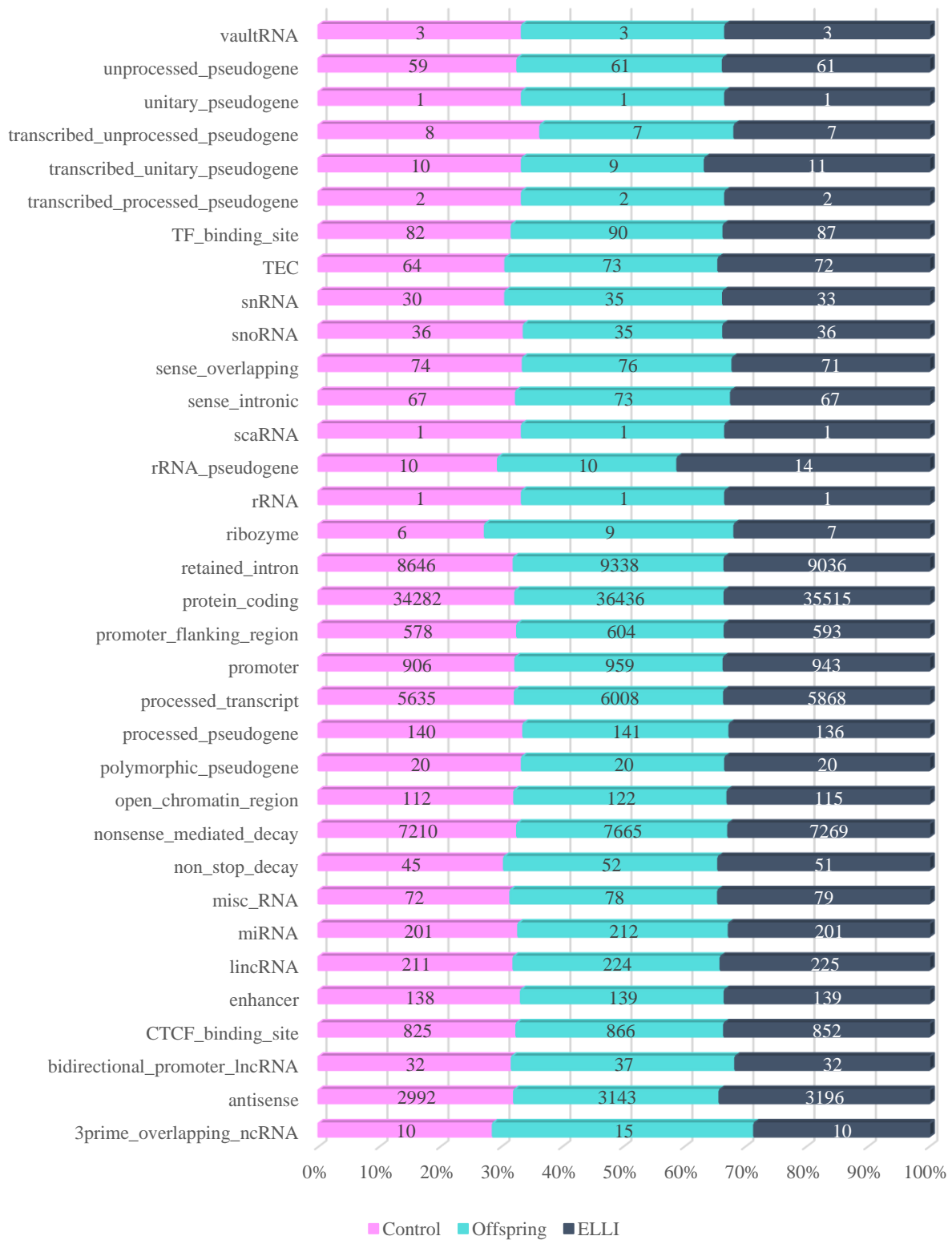

Supplement: Supplementary file 9 [file ACEL-19-e13216-s009.pdf]

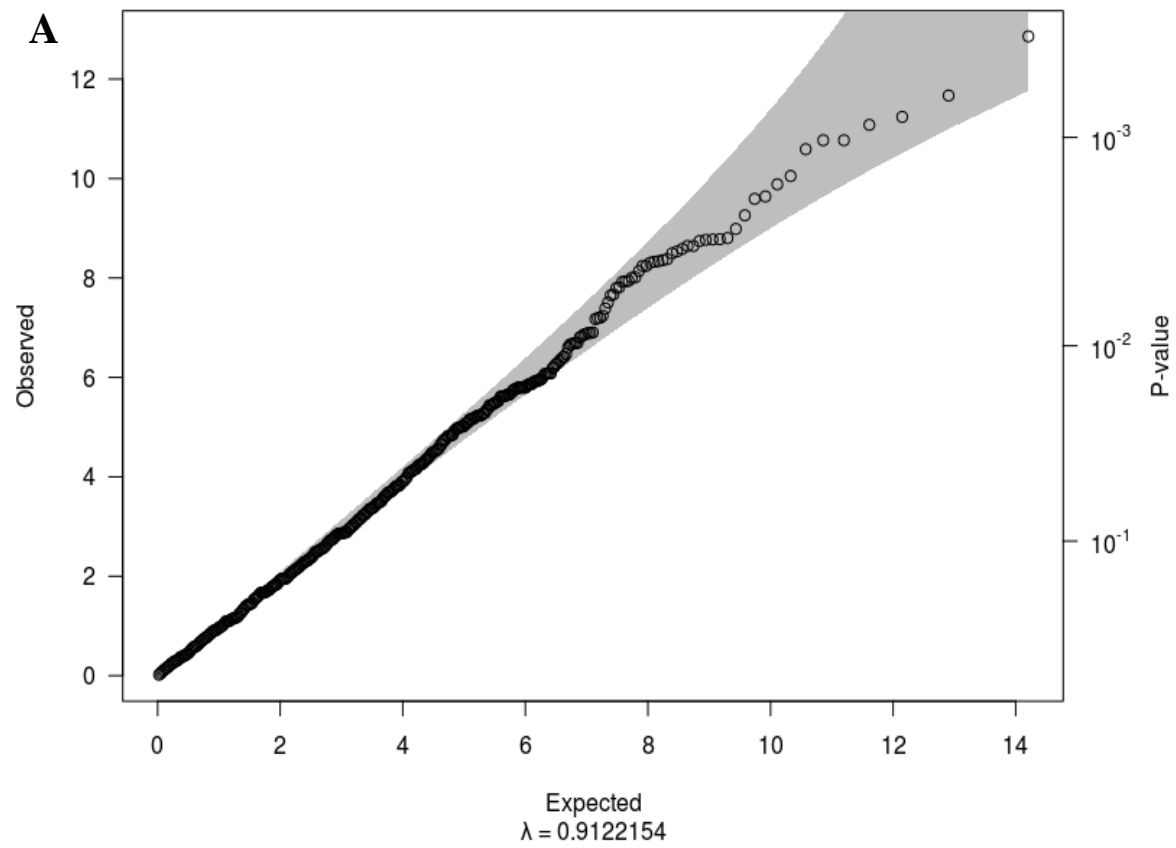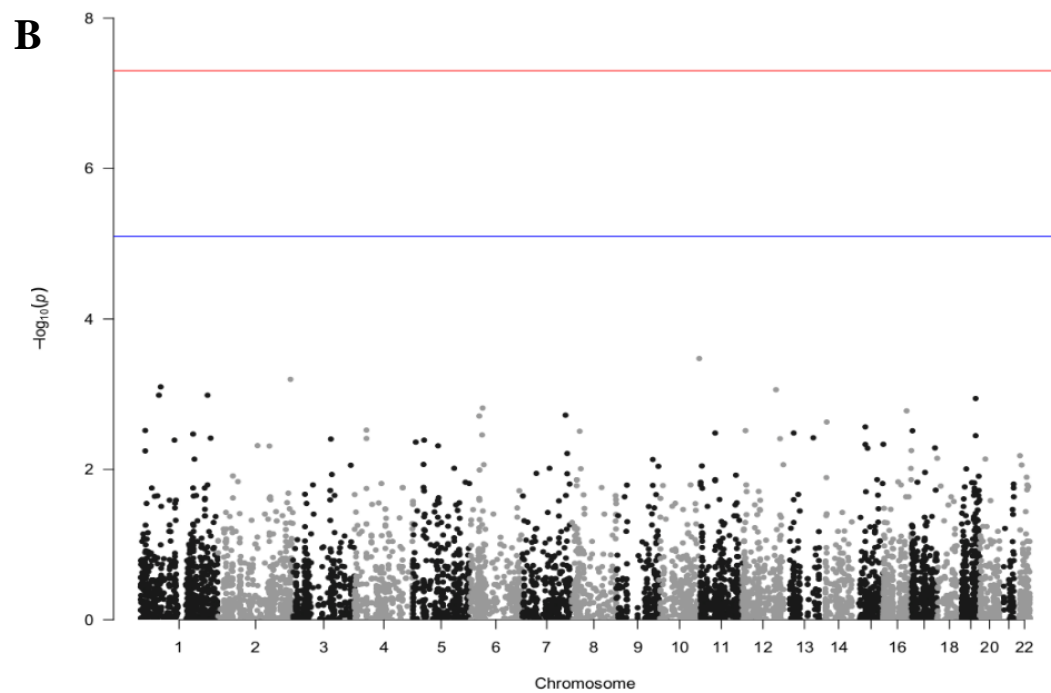

Supplement: Supplementary file 10 [file ACEL-19-e13216-s010.pdf]

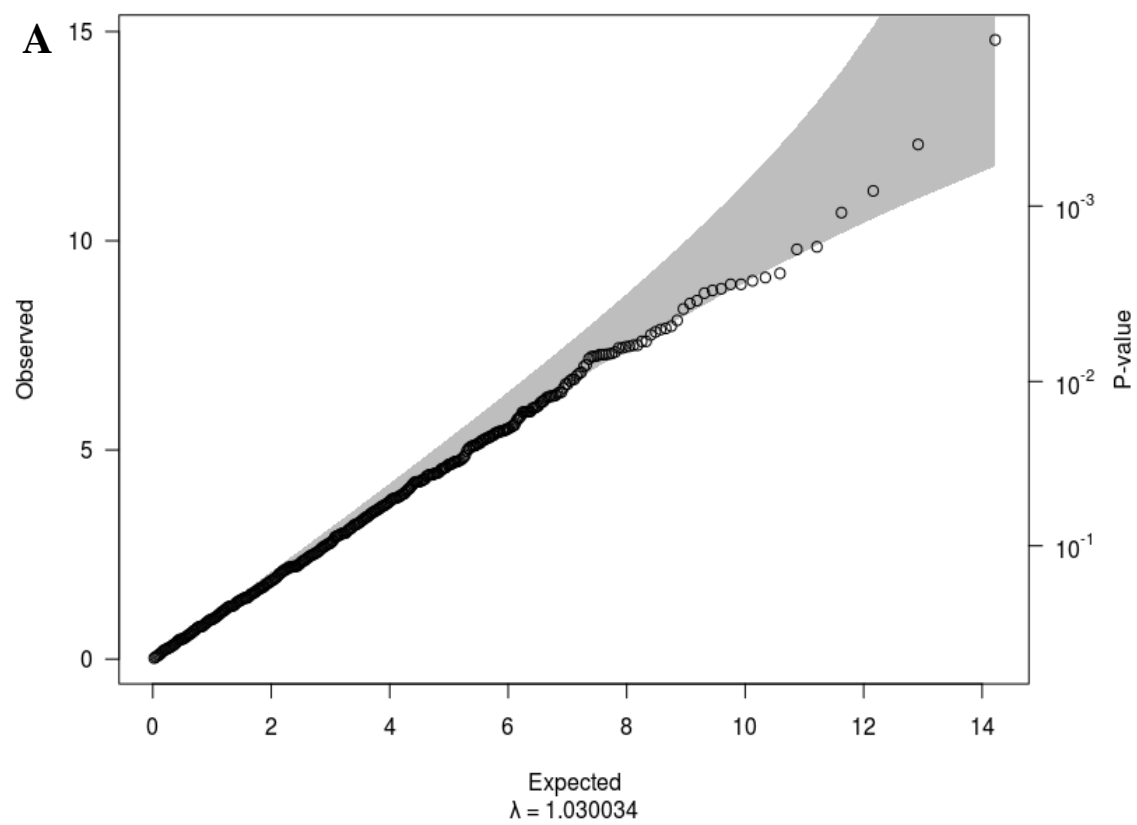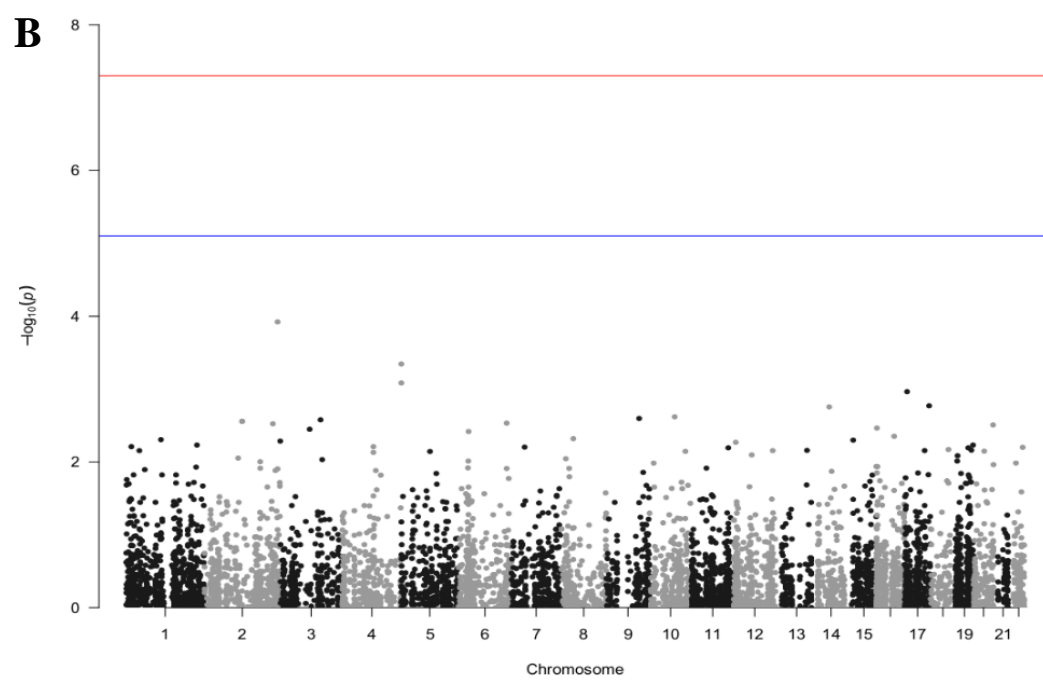

Supplement: Supplementary file 11 [file ACEL-19-e13216-s011.pdf]

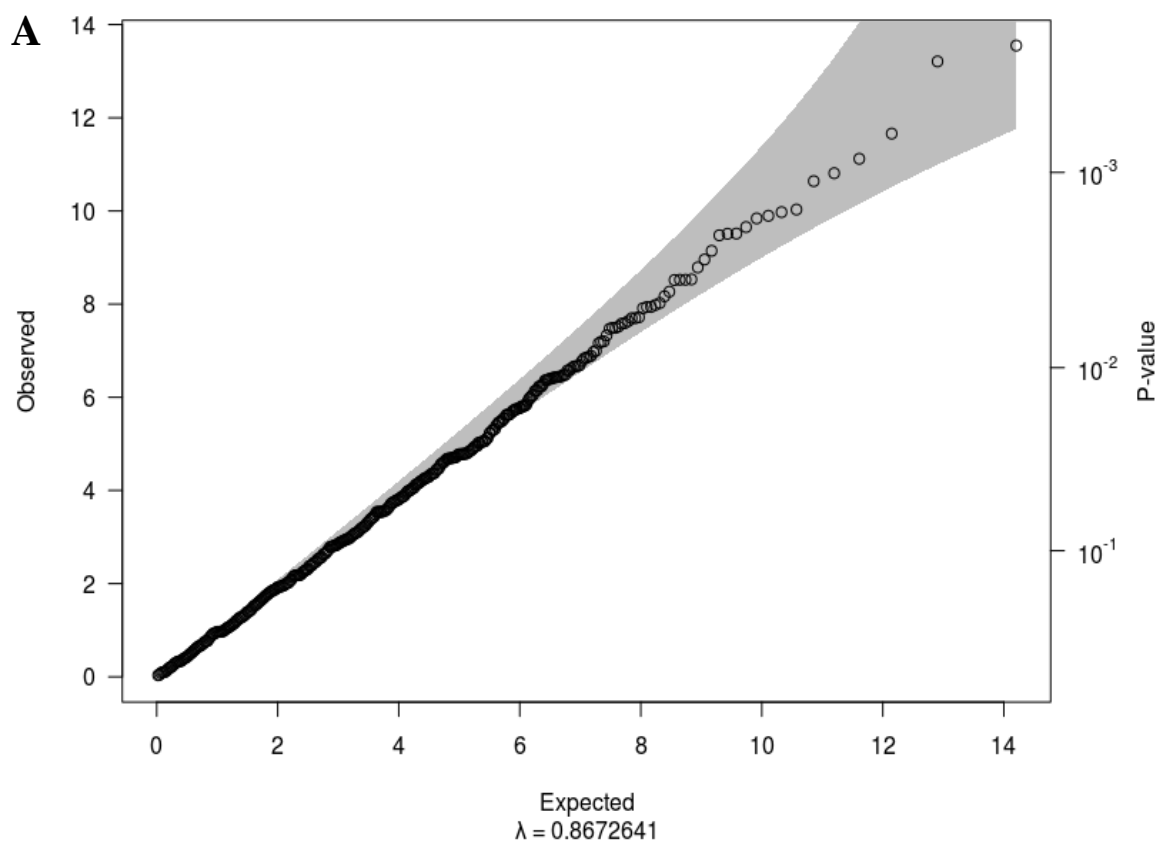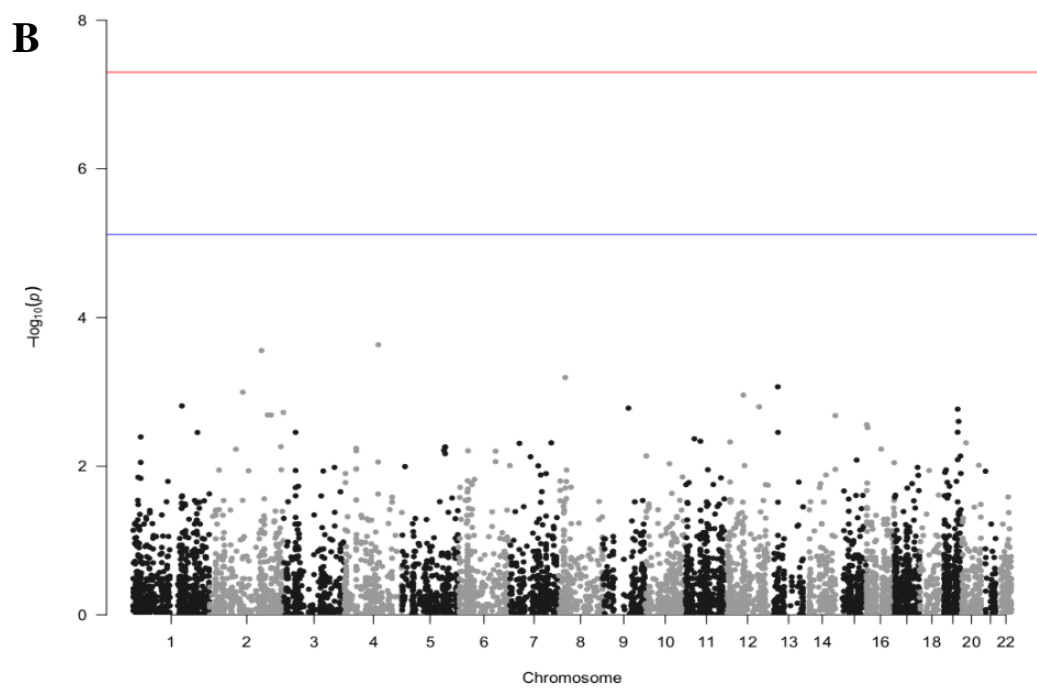

Supplement: Supplementary file 12 [file ACEL-19-e13216-s012.pdf]

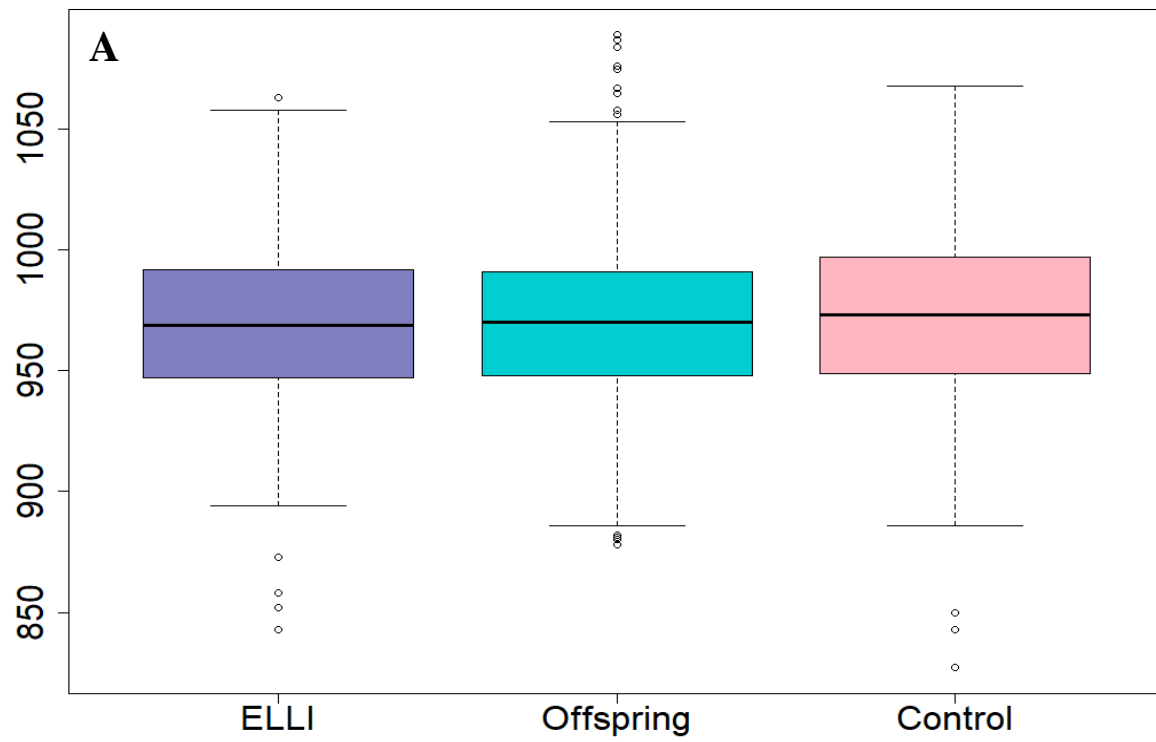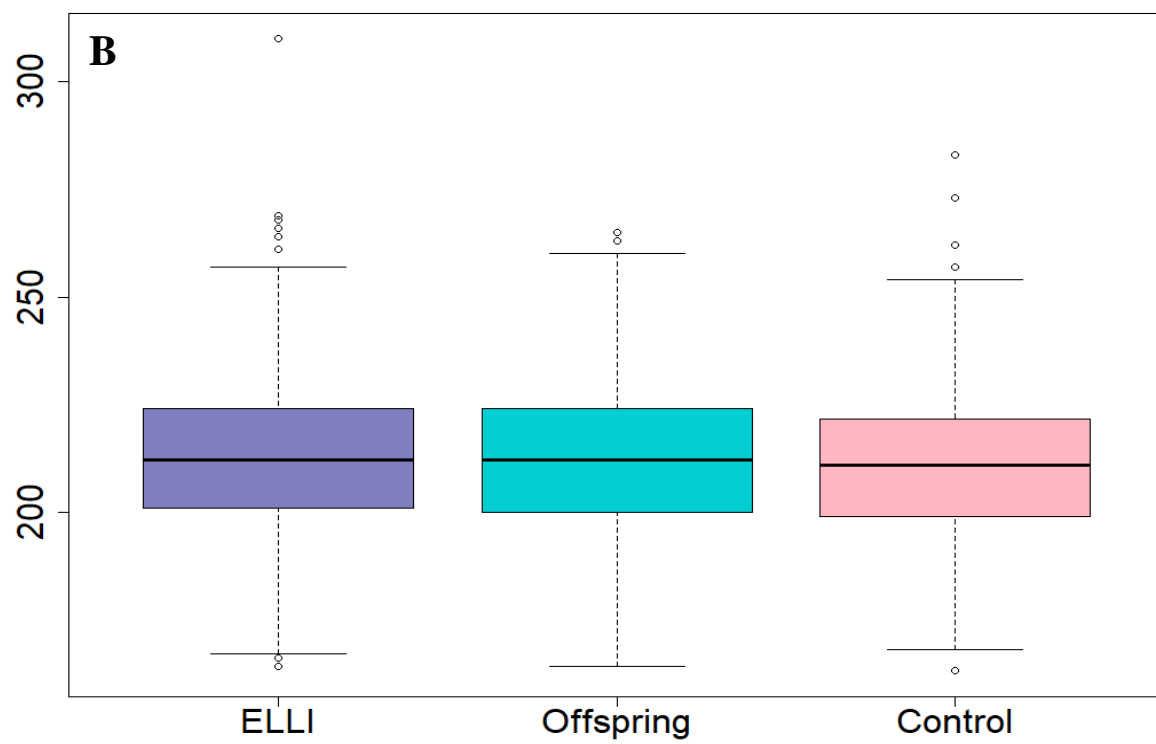

Supplement: Supplementary file 13 [file ACEL-19-e13216-s013.pdf]

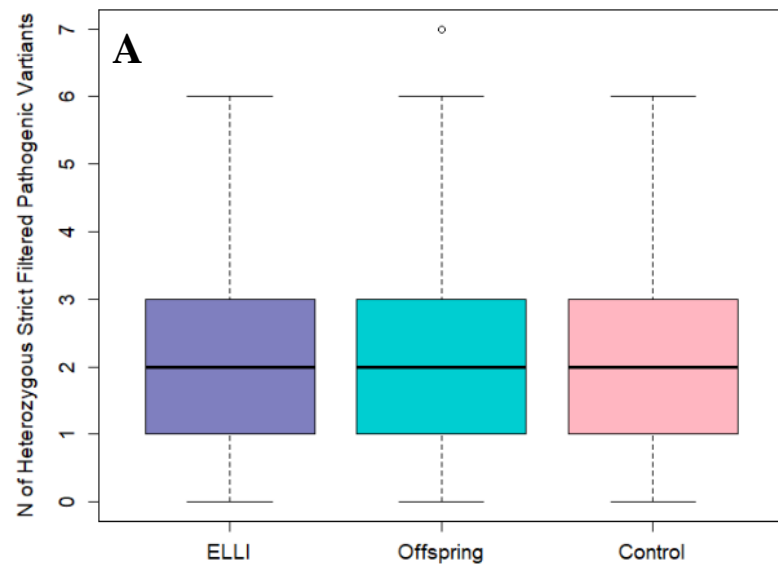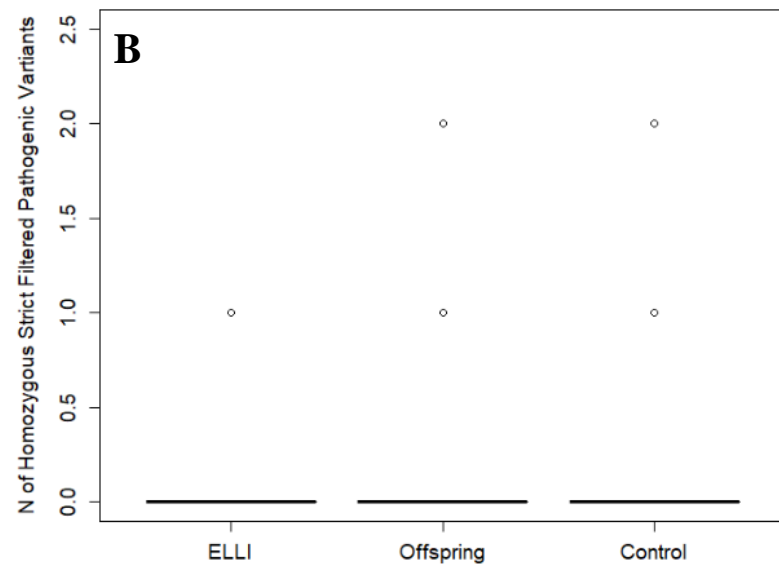

Supplement: Supplementary file 14 [file ACEL-19-e13216-s014.pdf]

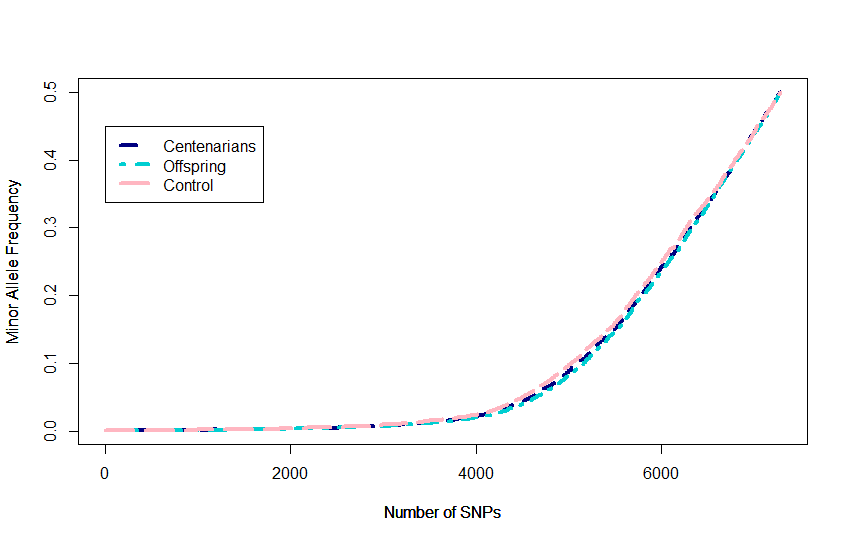

Supplement: Supplementary file 15 [file ACEL-19-e13216-s015.tiff]

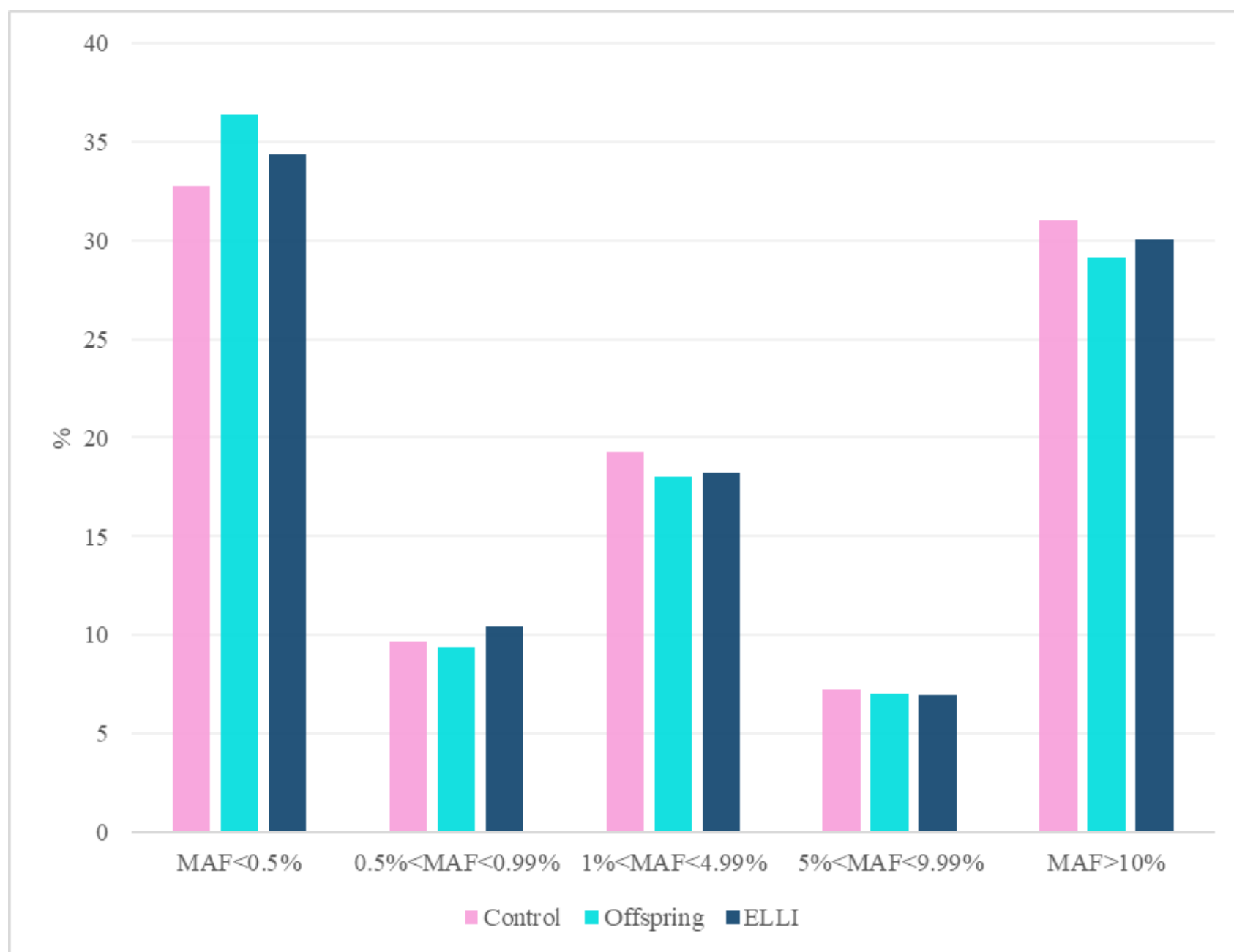

Supplement: Supplementary file 16 [file ACEL-19-e13216-s016.pdf]

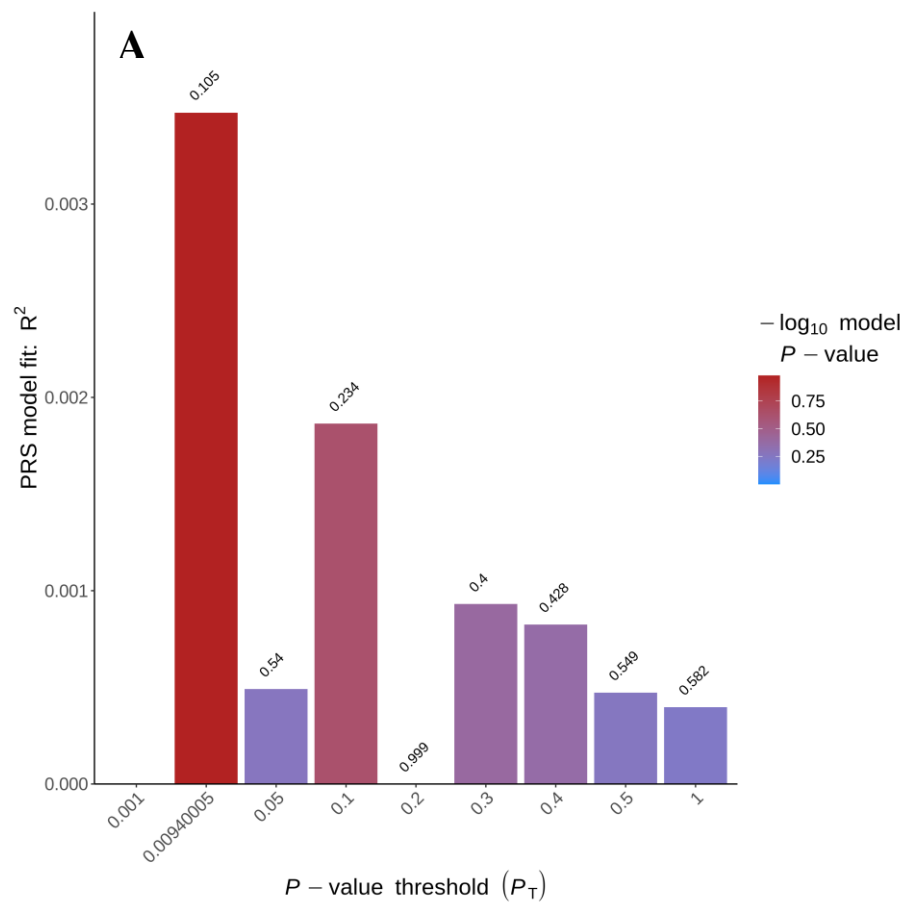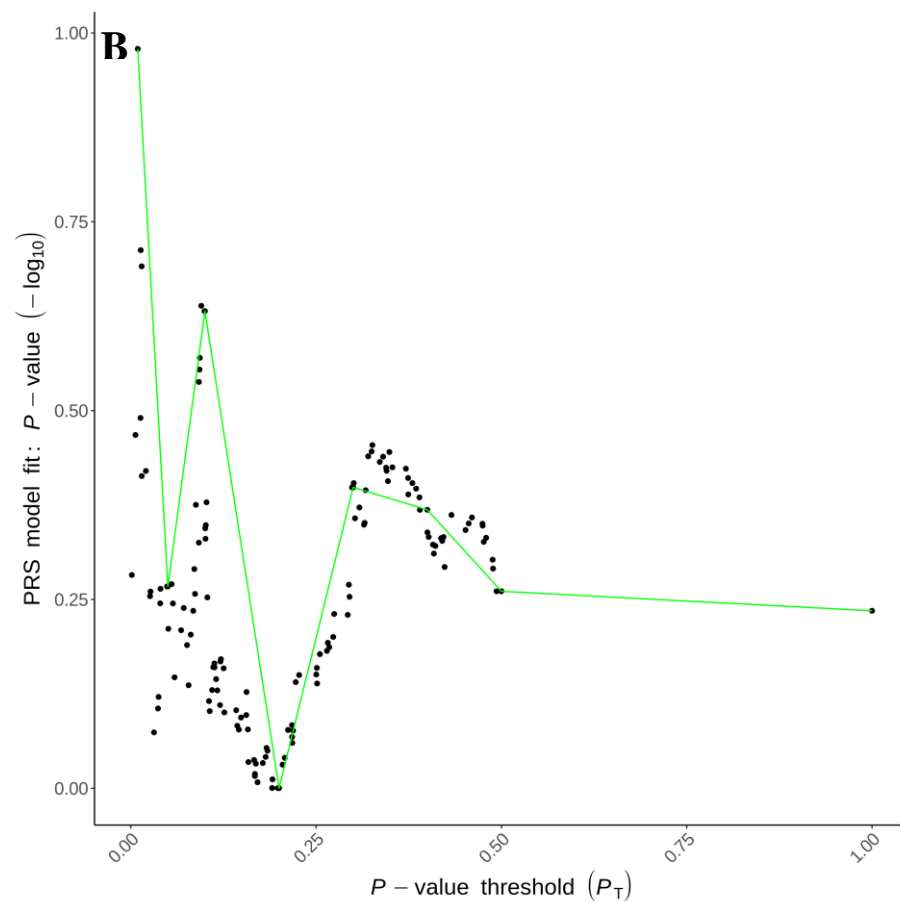

Supplement: Supplementary file 17 [file ACEL-19-e13216-s017.pdf]

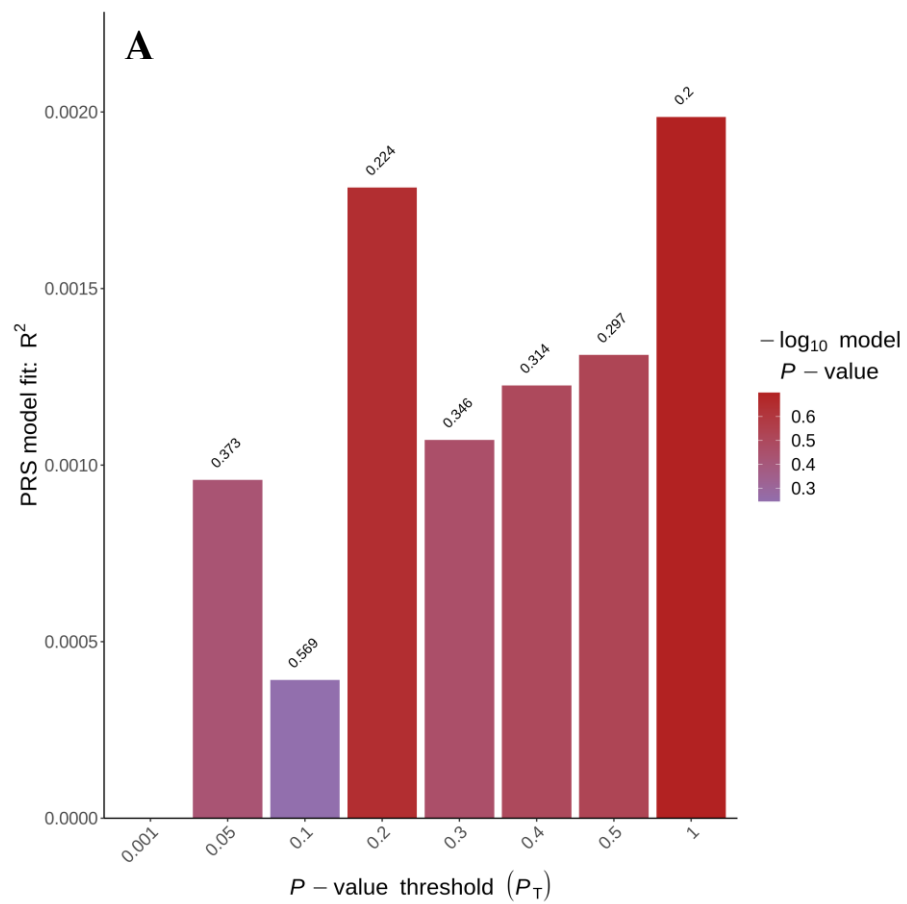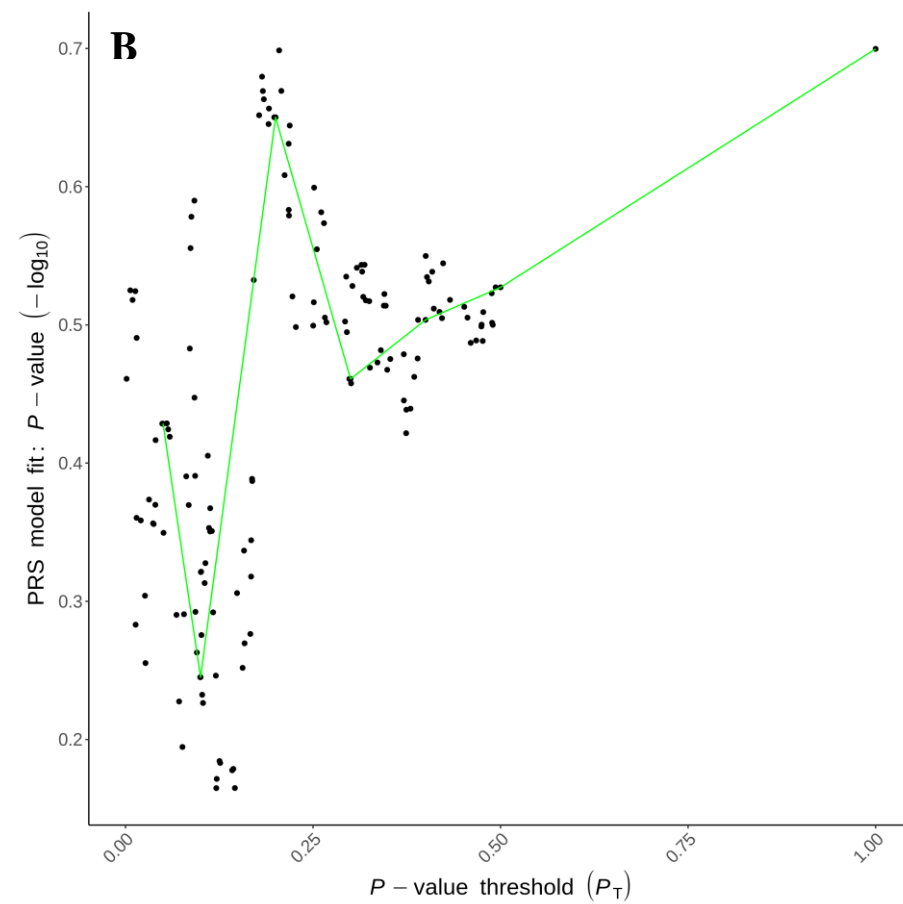

Supplement: Supplementary file 18 [file ACEL-19-e13216-s018.pdf]

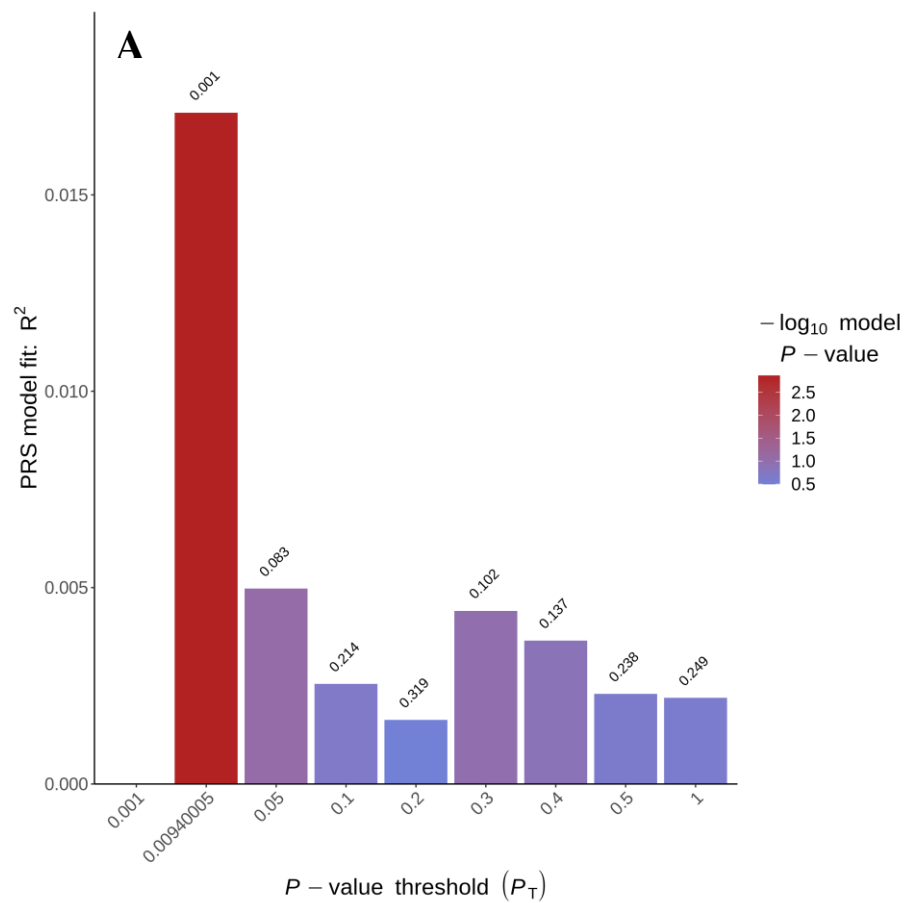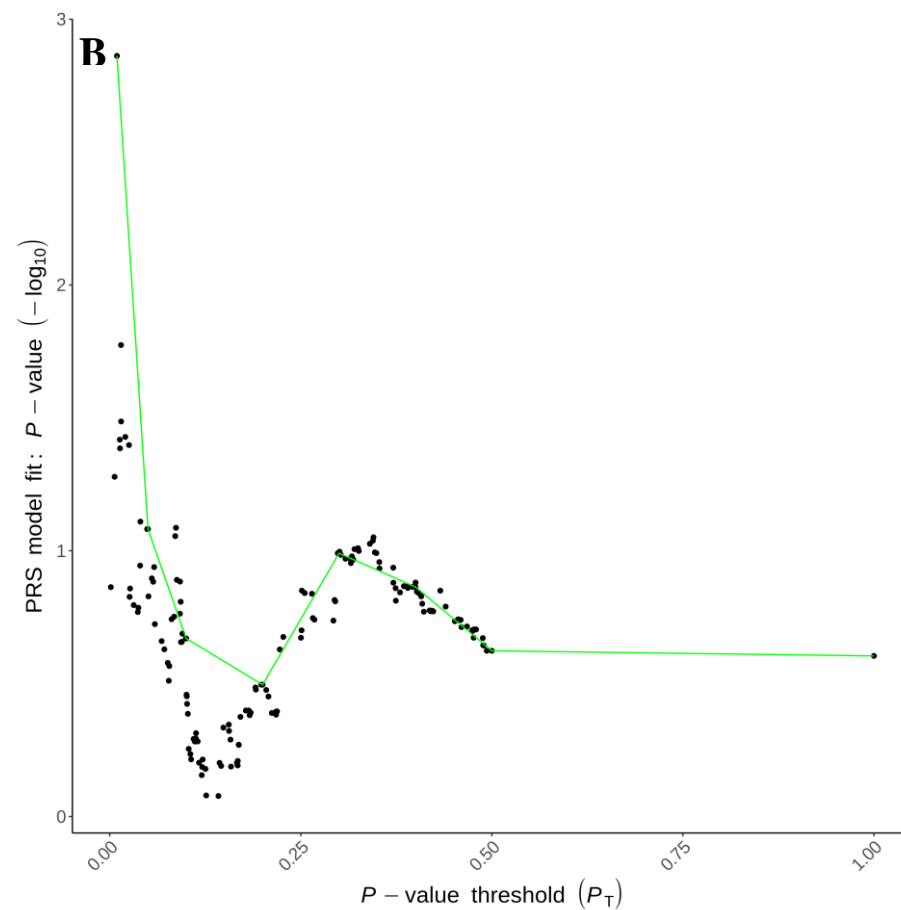

Supplement: Supplementary file 19 [file ACEL-19-e13216-s019.pdf]
