# Supplementary material for: Similar burden of pathogenic coding variants in exceptionally long‐lived individuals and individuals without exceptional longevity
Source: Aging Cell. 2020 Aug 29;19(10):e13216. doi: 10.1111/acel.13216 (PMC7576295; doi:10.1111/acel.13216)
Supplement: Supplementary file 6 [file ACEL-19-e13216-s006.pdf]

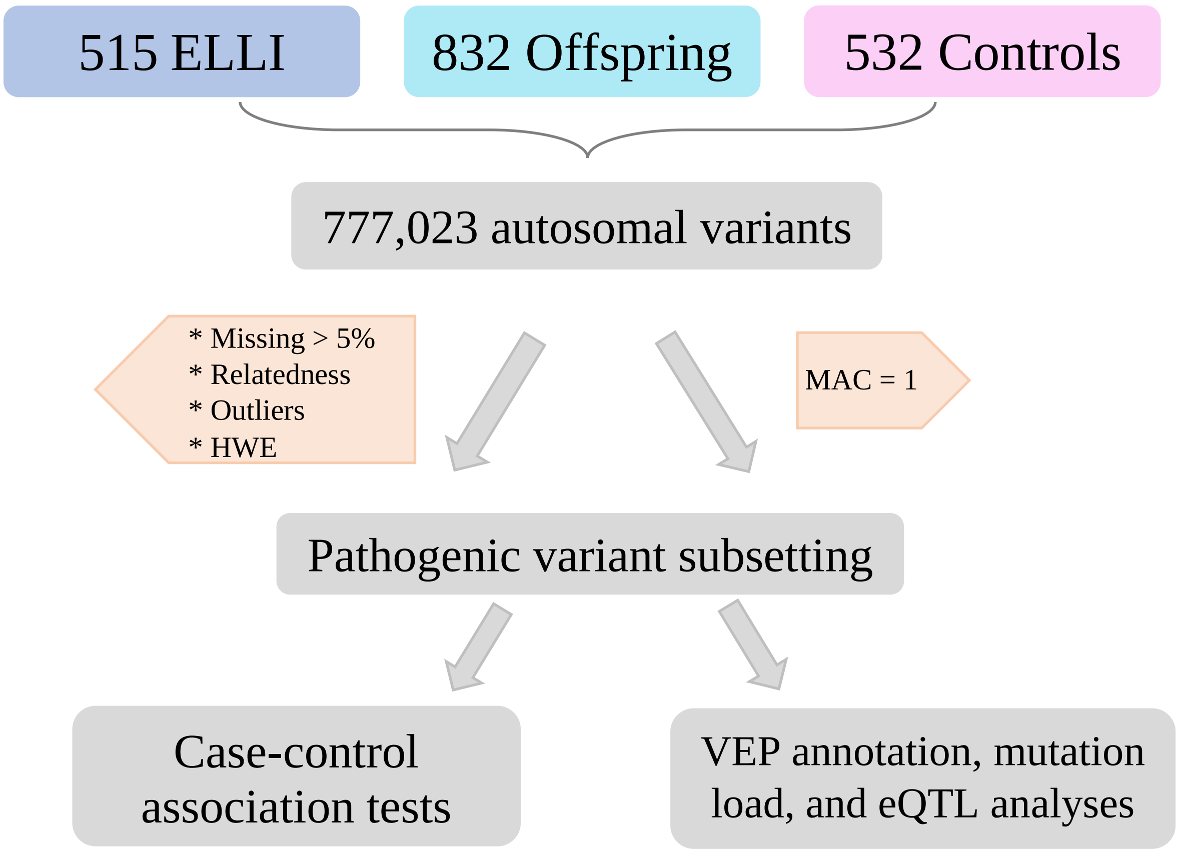
515 ELLI
832 Offspring
532 Controls
777,023 autosomal variants
* Missing > 5%
* Relatedness
* Outliers
* HWE
MAC = 1
Pathogenic variant subsetting
Case-control association tests
VEP annotation, mutation load, and eQTL analyses
