# Supplementary material for: Similar burden of pathogenic coding variants in exceptionally long‐lived individuals and individuals without exceptional longevity
Source: Aging Cell. 2020 Aug 29;19(10):e13216. doi: 10.1111/acel.13216 (PMC7576295; doi:10.1111/acel.13216)
Supplement: Supplementary file 8 [file ACEL-19-e13216-s008.pdf]

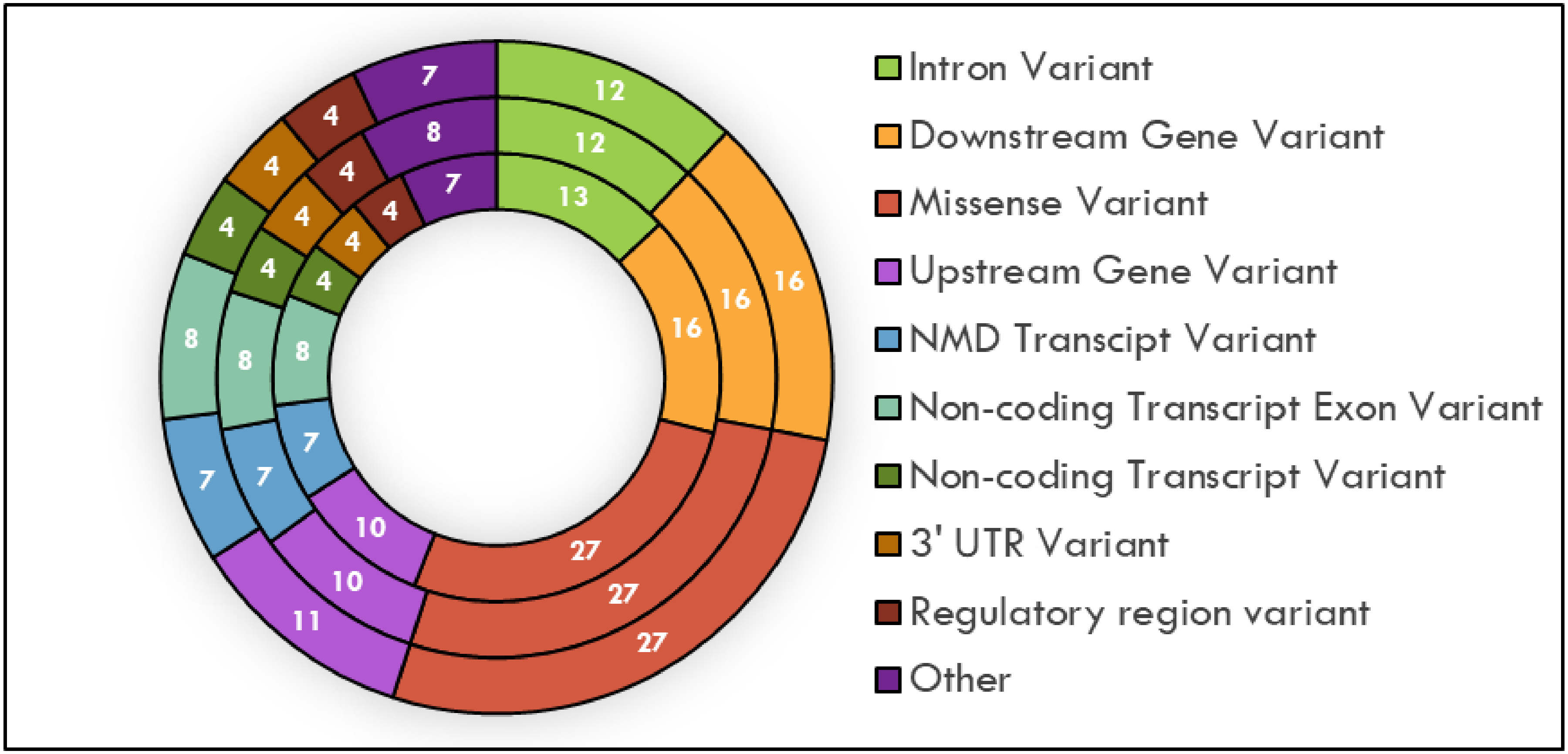

12
12
13
16
16
16
27
27
27
10
10
11
7
7
7
8
8
8
4
4
4
4
4
4
4
4
4
7
8
7
Intron Variant
Downstream Gene Variant
Missense Variant
Upstream Gene Variant
NMD Transcipt Variant
Non-coding Transcript Exon Variant
Non-coding Transcript Variant
3' UTR Variant
Regulatory region variant
Other
